# Supplementary figures and images for: Short- and long-range cis interactions between integrated HPV genomes and cellular chromatin dysregulate host gene expression in early cervical carcinogenesis
Source: PLoS Pathog. 2021 Aug 25;17(8):e1009875. doi: 10.1371/journal.ppat.1009875 (PMC8439666; doi:10.1371/journal.ppat.1009875)

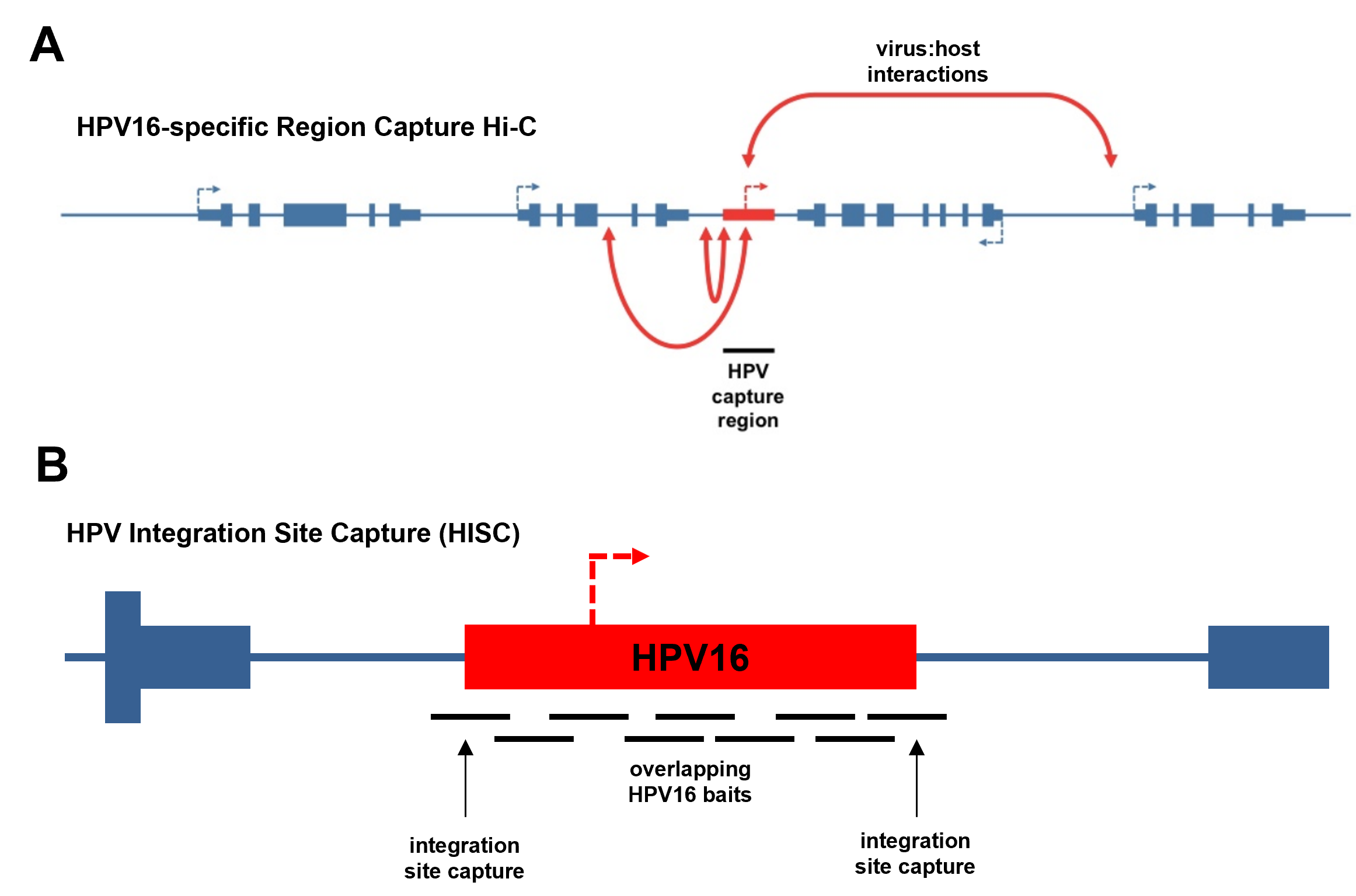

Supplement: S1 Fig — (A) HPV16-specific baits (consolidated pictorially as black line) are used to isolate both short- and long-range interactions (red double headed arrow) between a capture region (integrated HPV16 genome) and the host genome. (B) HPV16-specific baits (black lines) are used to enrich HPV16:host breakpoints from an ‘undigested’ sequencing library. (blue indicates host chromosome, genes and promoters.) (TIF) [file ppat.1009875.s001.tif]

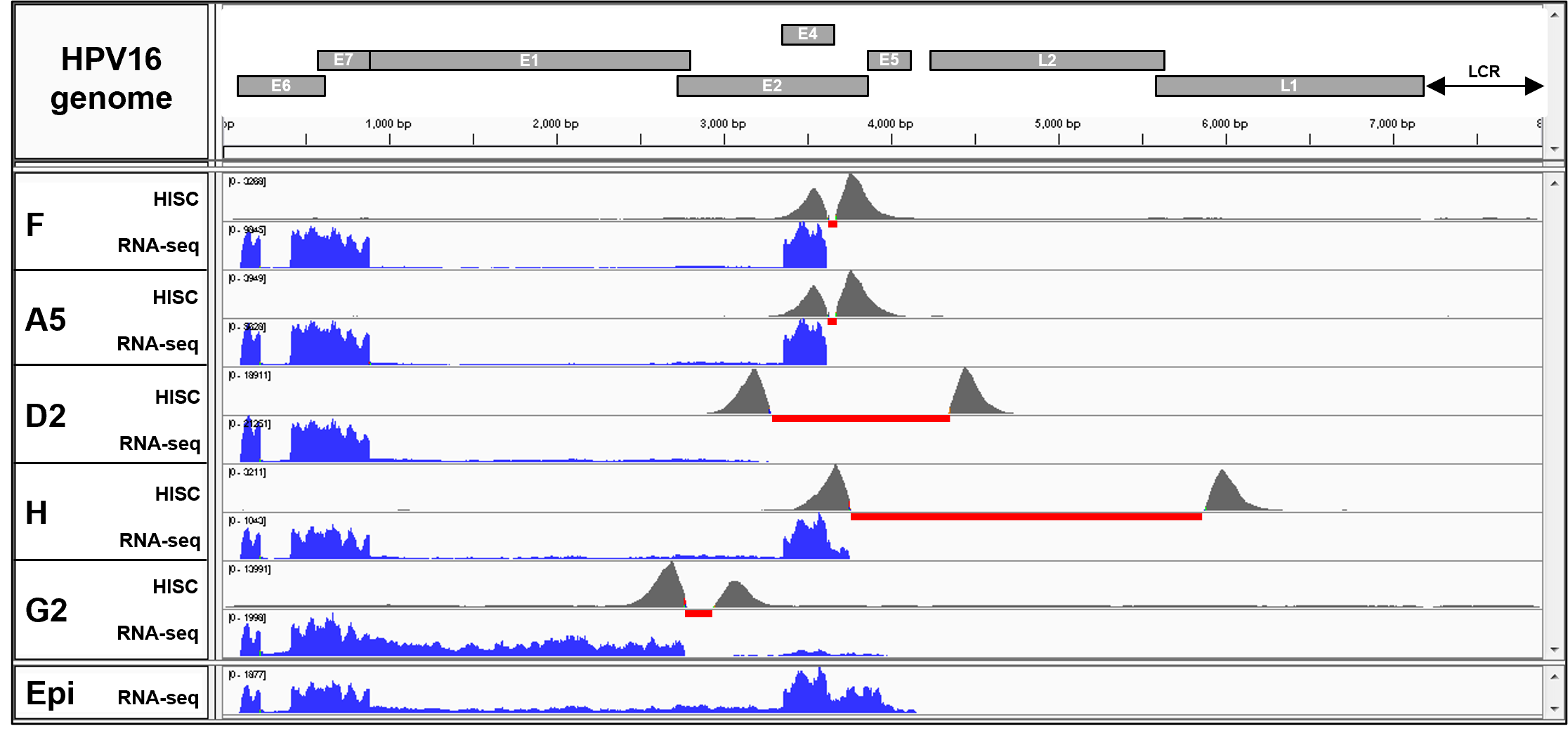

Supplement: S2 Fig — Aligned to a cartoon of the HPV16 genome are DNA read peaks from HISC analysis (grey peaks) determining breakpoints and deleted region of the HPV16 genome (red underline) as well as RNA read peaks (blue) denoting transcription across the virus genome for each of the W12 integrant clones used in the study along with RNA reads for the parental, episomal W12 Par1 (Epi) cell line for comparison. (TIF) [file ppat.1009875.s002.tif]

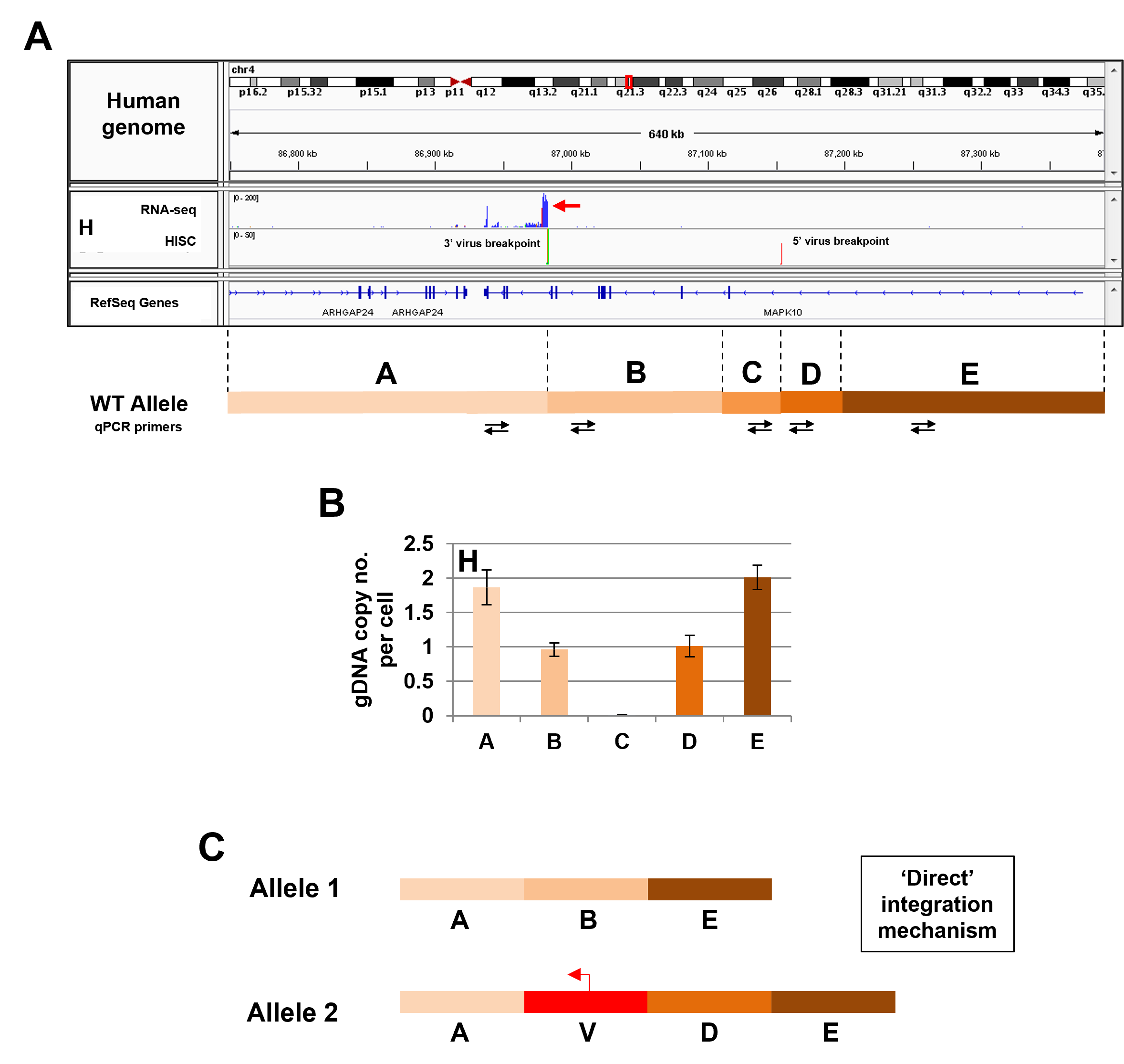

Supplement: S3 Fig — (A) RNA-seq data (blue peaks) showing transcription from host sequences driven by the integrant HPV16 genome (red arrow indicates the direction of which HPV16-host read-through transcription occurs through breakpoint) and HPV Integration Site Capture (HISC) data (multi-coloured peaks due to base calls) verifying virus-host breakpoints on the host genome. Wild-type allele regions indicated below with approximate location of qPCR primer sites. (B) qPCR of genomic DNA regions to determine copy number after HPV16 genome integration in clone H. (C) Determination of arrangement of gDNA sections after HPV16 genome integration through ‘direct’ mechanism, causing deletion of regions including homozygous deletion of region C. Virus copy number (V, 1) from Scarpini et al., 2014. Not to scale. (TIF) [file ppat.1009875.s003.tif]

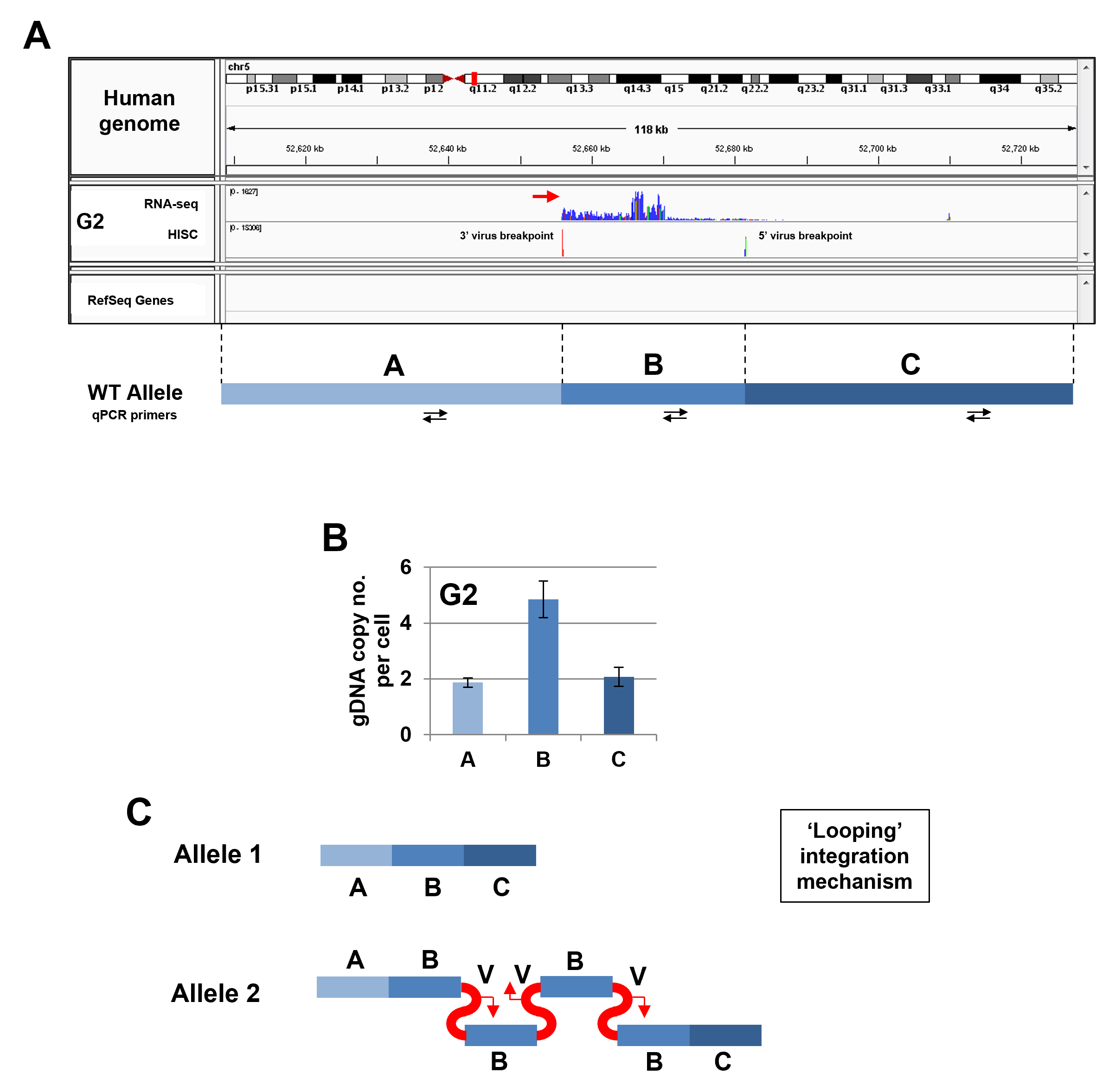

Supplement: S4 Fig — (A) RNA-seq data (blue peaks) showing transcription from host sequences driven by the integrant HPV16 genome (red arrow indicates the direction of which HPV16-host read-through transcription occurs through breakpoint) and HPV Integration Site Capture (HISC) data (multi-coloured peaks due to base calls) verifying virus-host breakpoints on the host genome. Wild-type allele regions indicated below with approximate location of qPCR primer sites. (B) qPCR of genomic DNA regions to determine copy number after HPV16 genome integration in clone G2. (C) Determination of arrangement of gDNA sections after HPV16 genome integration through ‘looping’ mechanism, amplifying region B. Virus copy number (V, 3) based on Scarpini et al., 2014. Not to scale. (TIF) [file ppat.1009875.s004.tif]

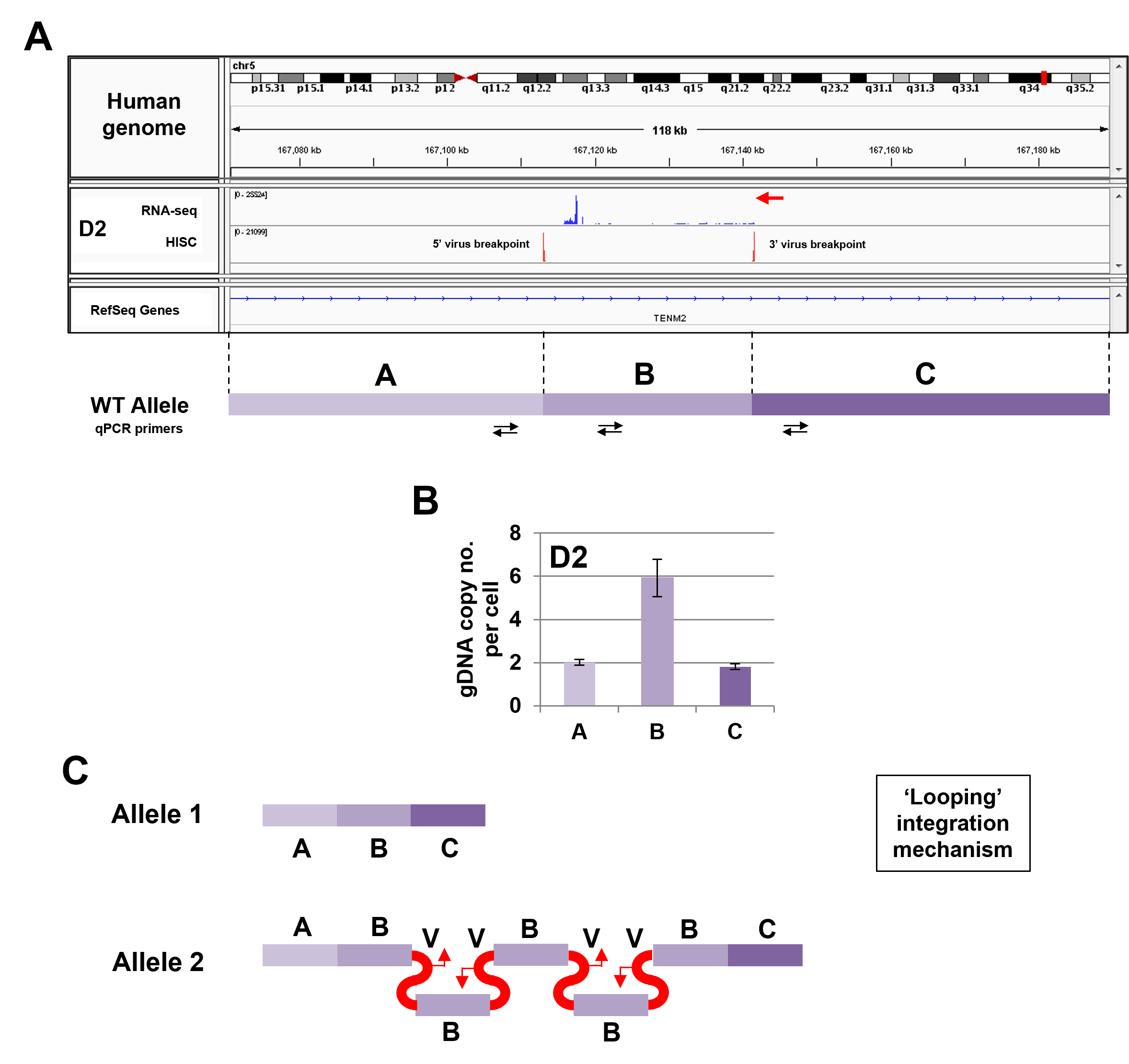

Supplement: S5 Fig — (A) RNA-seq data (blue peaks) showing transcription from host sequences driven by the integrant HPV16 genome (red arrow indicates the direction of which HPV16-host read-through transcription occurs through breakpoint) and HPV Integration Site Capture (HISC) data (multi-coloured peaks due to base calls) verifying virus-host breakpoints on the host genome. Wild-type allele regions indicated below with approximate location of qPCR primer sites. (B) qPCR of genomic DNA regions to determine copy number after HPV16 genome integration in clone D2. (C) Determination of arrangement of gDNA sections after HPV16 genome integration through ‘looping’ mechanism, amplifying region B. Virus copy number (V, 4) from Scarpini et al., 2014. Not to scale. (TIF) [file ppat.1009875.s005.tif]

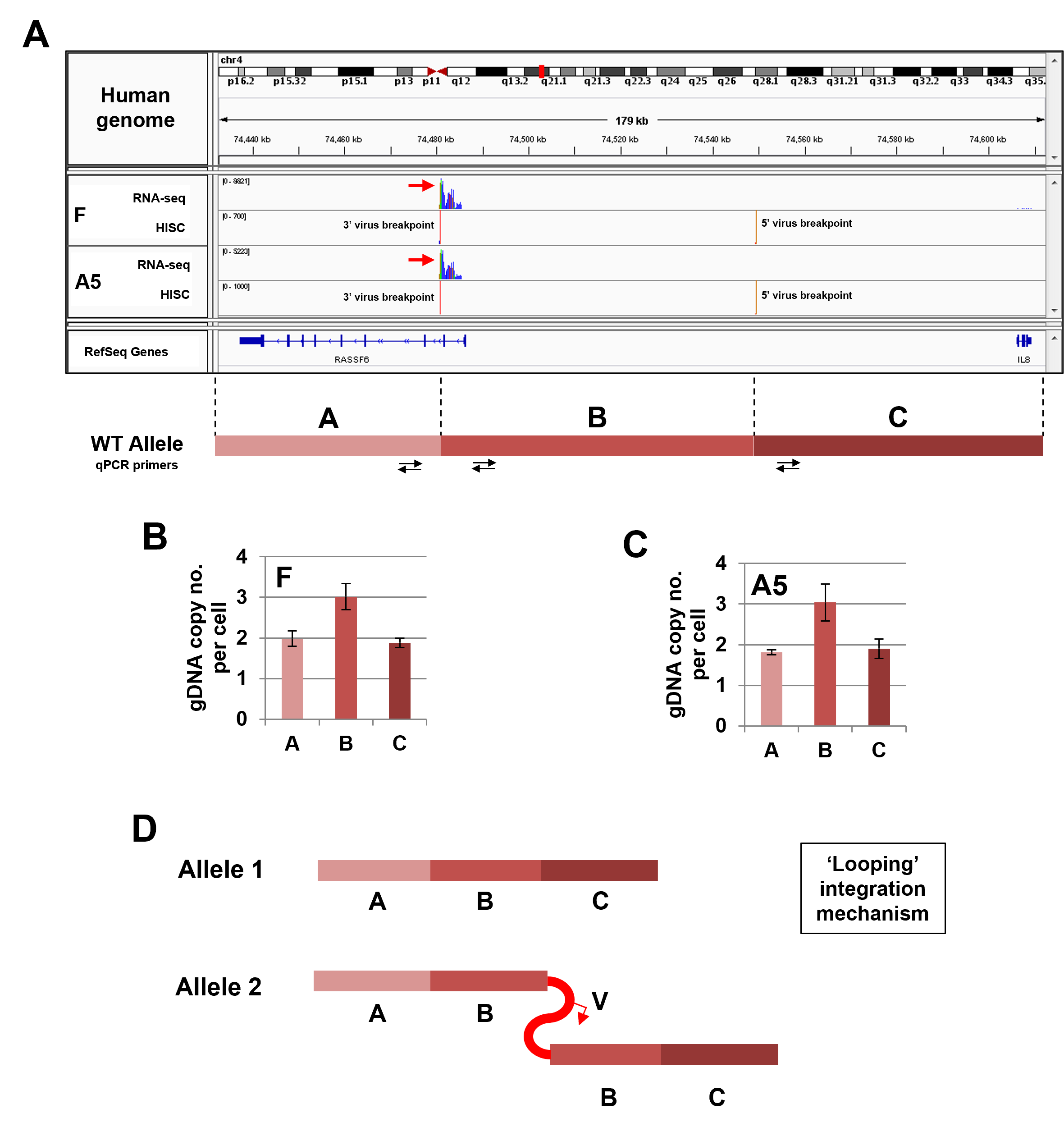

Supplement: S6 Fig — (A) RNA-seq data (blue peaks) showing transcription from host sequences driven by the integrant HPV16 genome (red arrow indicates the direction of which HPV16-host read-through transcription occurs through breakpoint) and HPV Integration Site Capture (HISC) data (multi-coloured peaks due to base calls) verifying virus-host breakpoints on the host genome. Wild-type allele regions indicated below with approximate location of qPCR primer sites. qPCR of genomic DNA regions to determine copy number after HPV16 genome integration in clones (B) F and (C) A5. (D) Determination of arrangement of gDNA sections after HPV16 genome integration through ‘looping’ mechanism, amplifying region B. Virus copy number (V, 1) from Scarpini et al., 2014. Not to scale. (TIF) [file ppat.1009875.s006.tif]

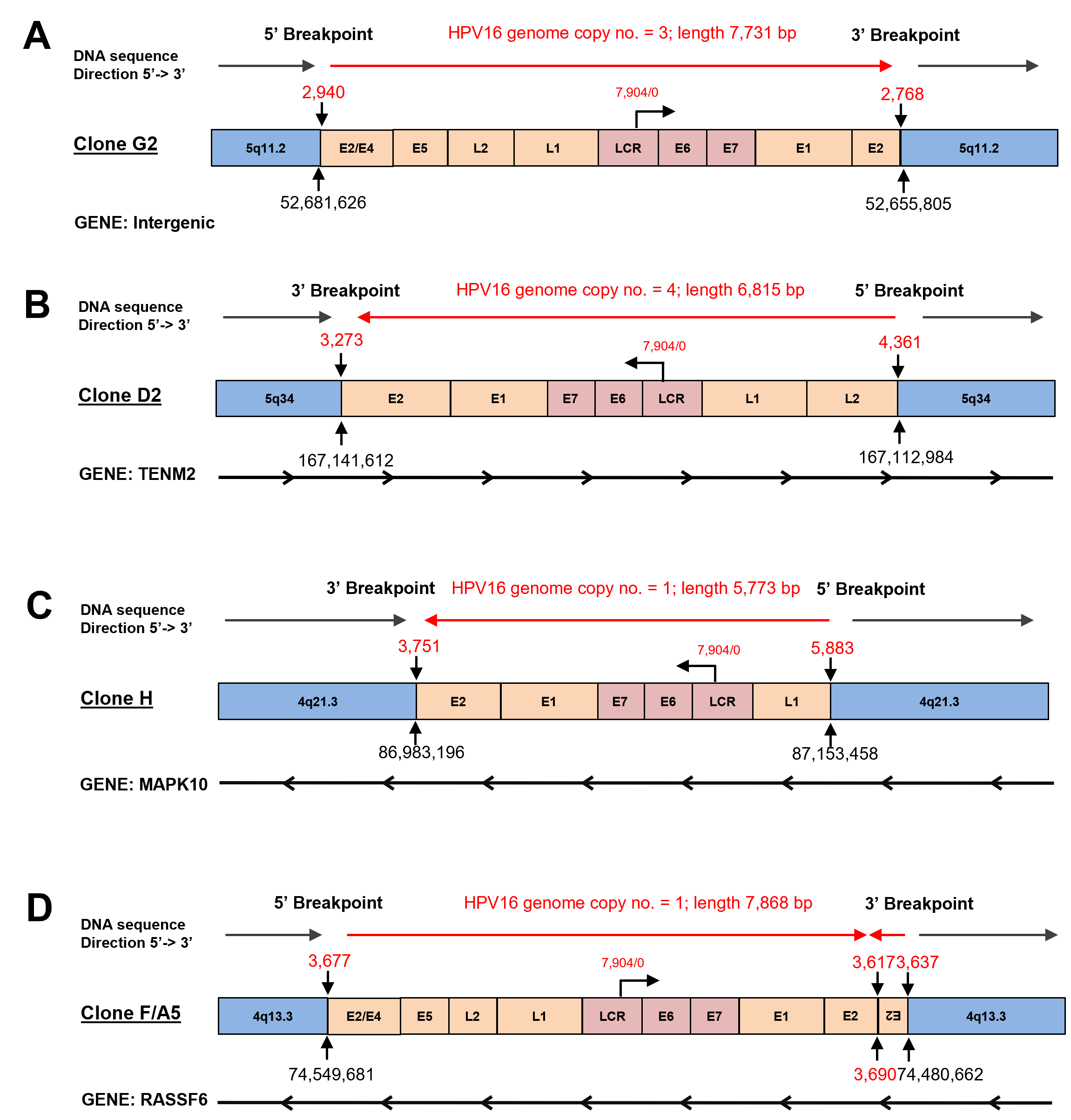

Supplement: S7 Fig — In all schematics, host chromosomal DNA is shown in blue and the orientation indicated by the grey arrow above (5’ to 3’). Integrated HPV16 DNA is shown in orange, with the viral oncogenes and long control region (LCR) highlighted in red, and the direction of transcription from the viral early promoter shown by an arrow from the LCR. The location of the viral breakpoint in base pairs is given above the junction, whereas the cellular DNA breakpoint in base pairs is given below the junction. The genome copy number and length of the integrated HPV16 genome is indicated in red above the schematic. When HPV16 has integrated into a host gene, the orientation of this gene is shown beneath the schematic in black. (A) W12 clone G2, (B) D2, (C) H and (D) W12 clones F and A5. (Virus genome copy number taken from Scarpini et al., 2014.) (TIF) [file ppat.1009875.s007.tif]

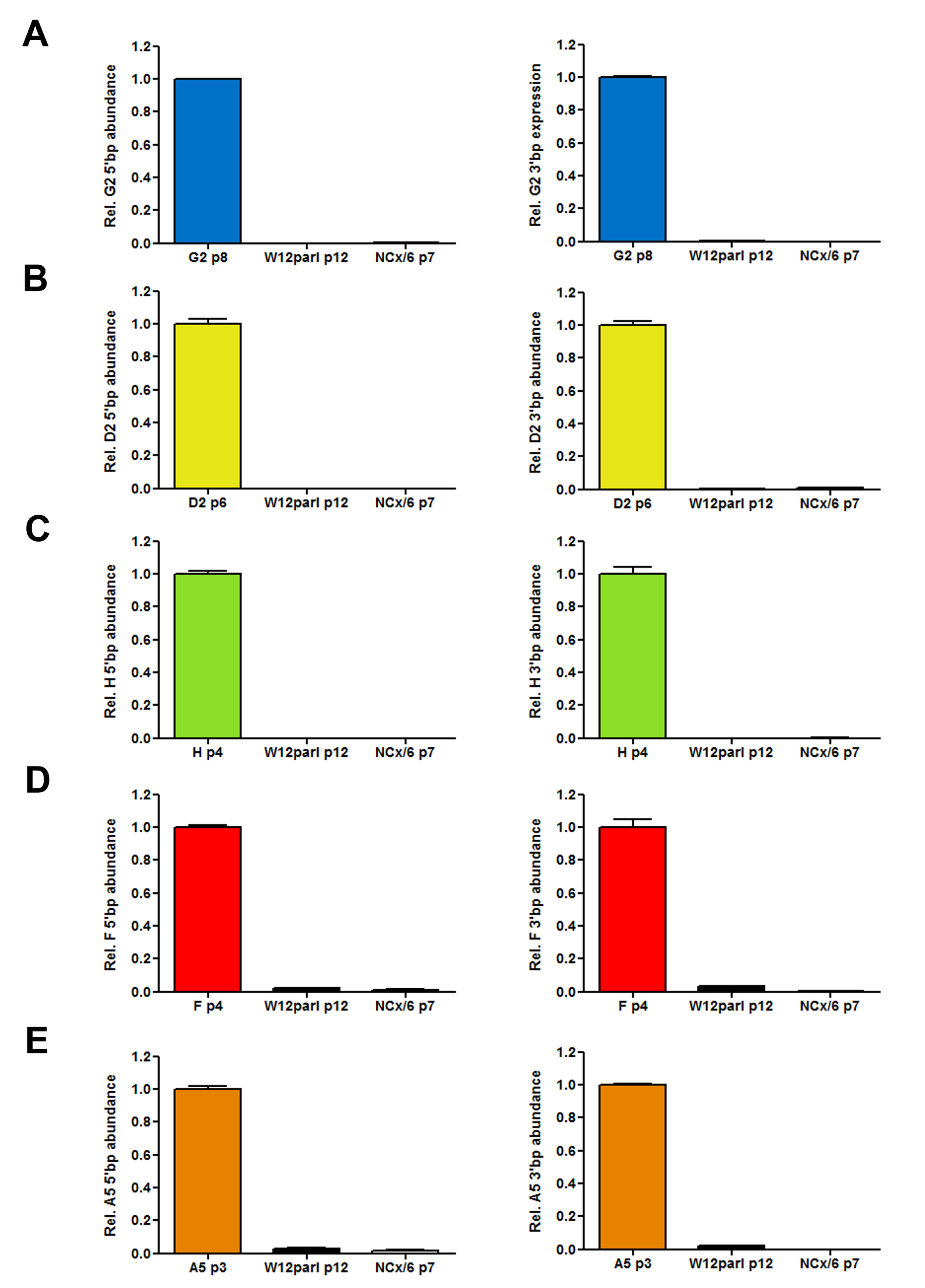

Supplement: S8 Fig — To confirm the virus-host breakpoints found in each W12 integrant cloned line, pairs of qPCR primers were designed to amplify the 5’ (left column) and 3’ (right column) breakpoints from genomic DNA samples for clones (A) G2, (B) D2, (C) H, (D) F and (E) A5 in comparison to the episomal (W12par1) cell line and HPV-negative cell line (NCx/6). (TIF) [file ppat.1009875.s008.tif]

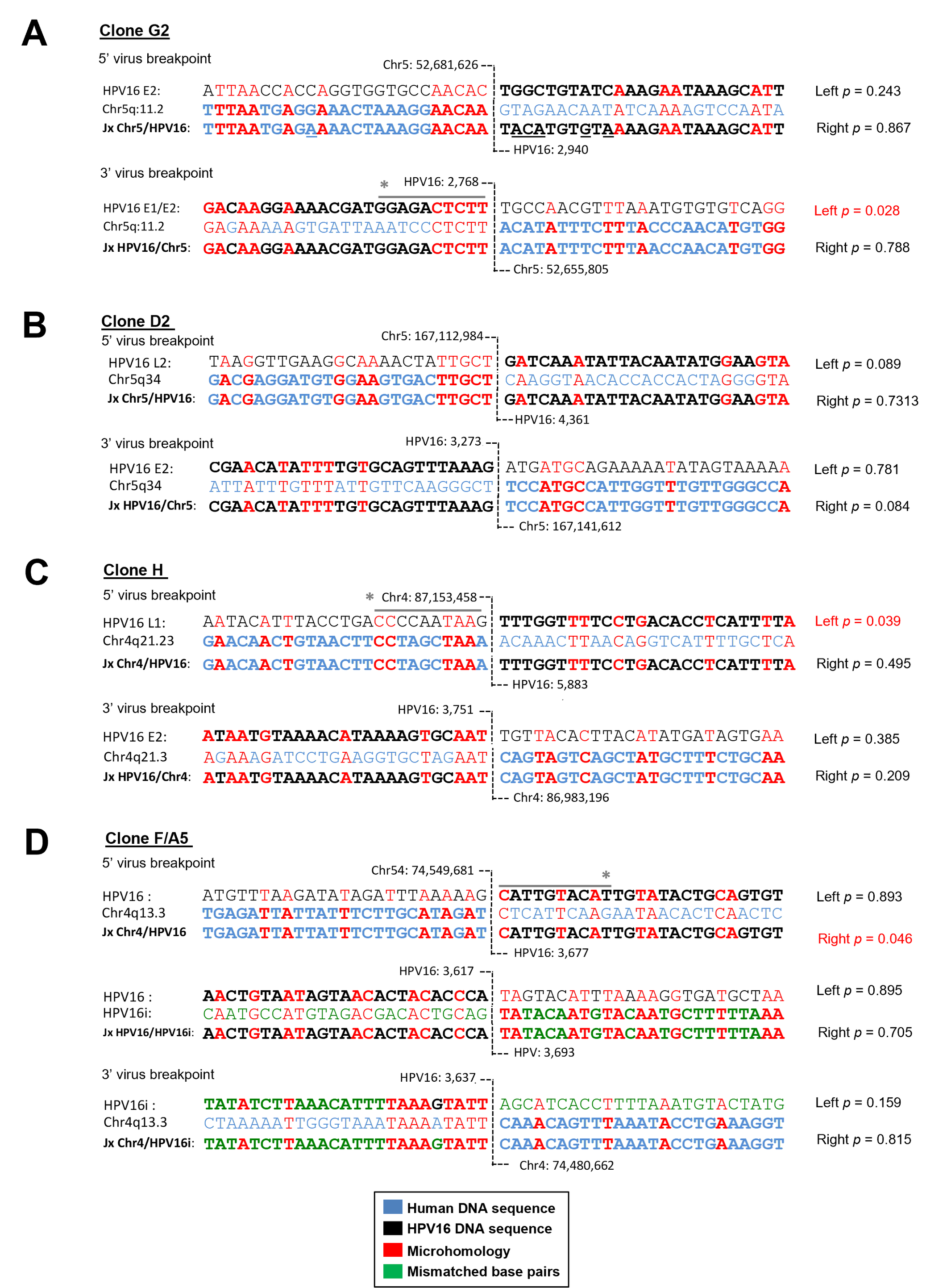

Supplement: S9 Fig — Figures show comparisons between the virus-host sequences obtained by Sanger sequencing and the normal host and HPV16 genomic sequences, 25 nucleotides either side of the breakpoint (indicated by a central dotted line). HPV16 DNA sequence = black, inverted HPV16 DNA sequence = green, human DNA sequence = blue, homologous nucleotides = red. Significant levels of microhomology between host and HPV16 sequences were calculated by comparing the homology seen at the 10 nt directly either side of the breakpoint compared to 1000 nt of extended sequence which was shuffled 10,000 times and are indicated by a line above appropriate sequences. *p<0.05 (highlighted in red). (TIF) [file ppat.1009875.s009.tif]

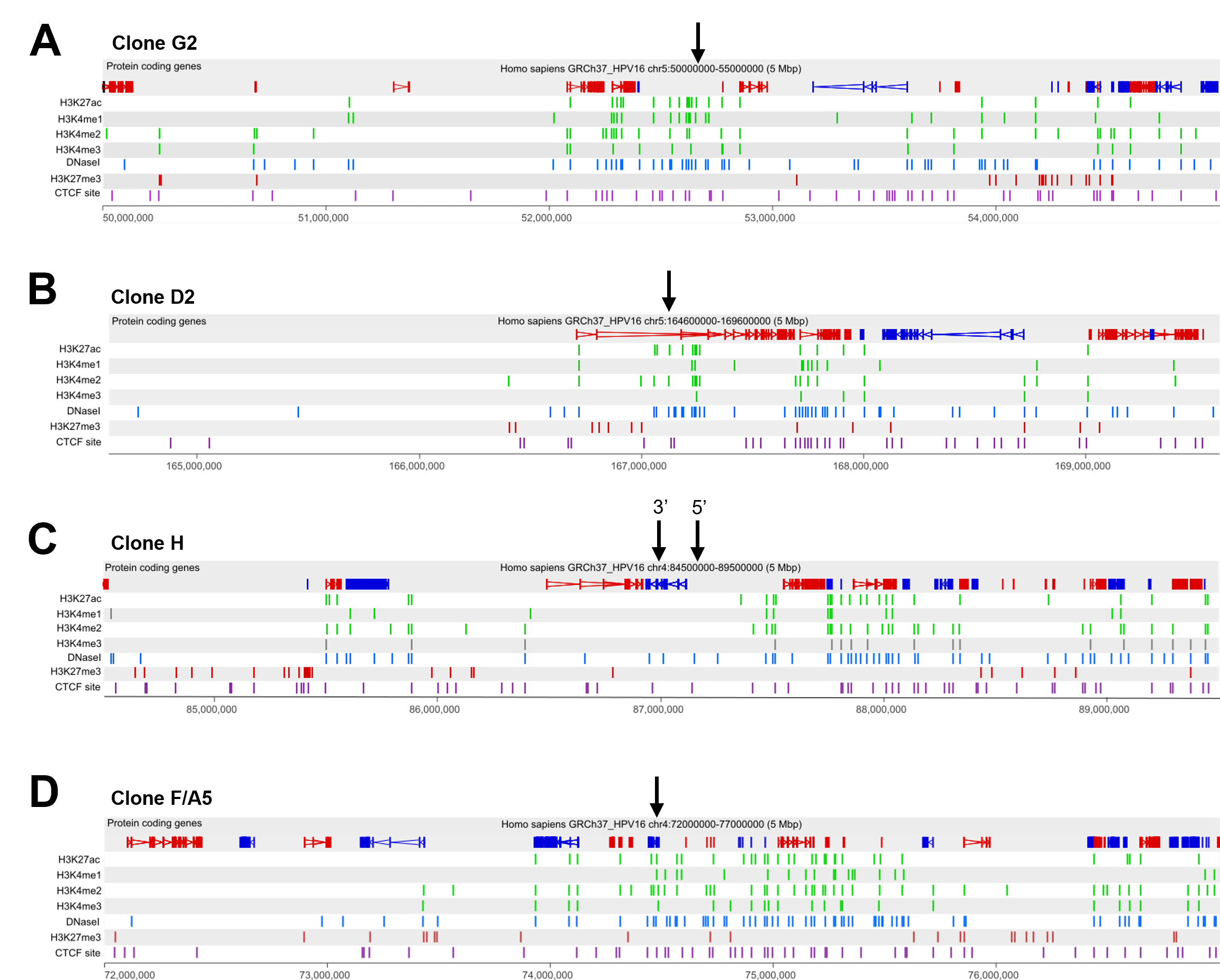

Supplement: S10 Fig — Each panel shows 5 Mb of the host genome across the integration loci for W12 clones (A) G2, (B) D2, (C) H, and (D) F/A5 with the virus integration site indicated by a black arrow. Protein coding genes are shown in the first track and the direction of each gene indicated by colour (red, forward; blue, reverse). ChIP-seq data from a normal human epidermal keratinocyte (NHEK, taken from ENCODE) cell line is aligned with the host genome. Post-translational histone modifications of host enhancers (H3K27ac, H3K4me1; green), active promoters (H3K4me2, H3K4me3; green), repressed chromatin (H3K27me3, red), DNaseI hypersensitivity sites (blue) and CTCF sites (purple) are shown. Coordinates presented for each clone are indicated at the top of each figure. W12 clone H has both 5’ and 3’ ends of the HPV16 genome identified due to the length of the host genomic deletion. (TIF) [file ppat.1009875.s010.tif]

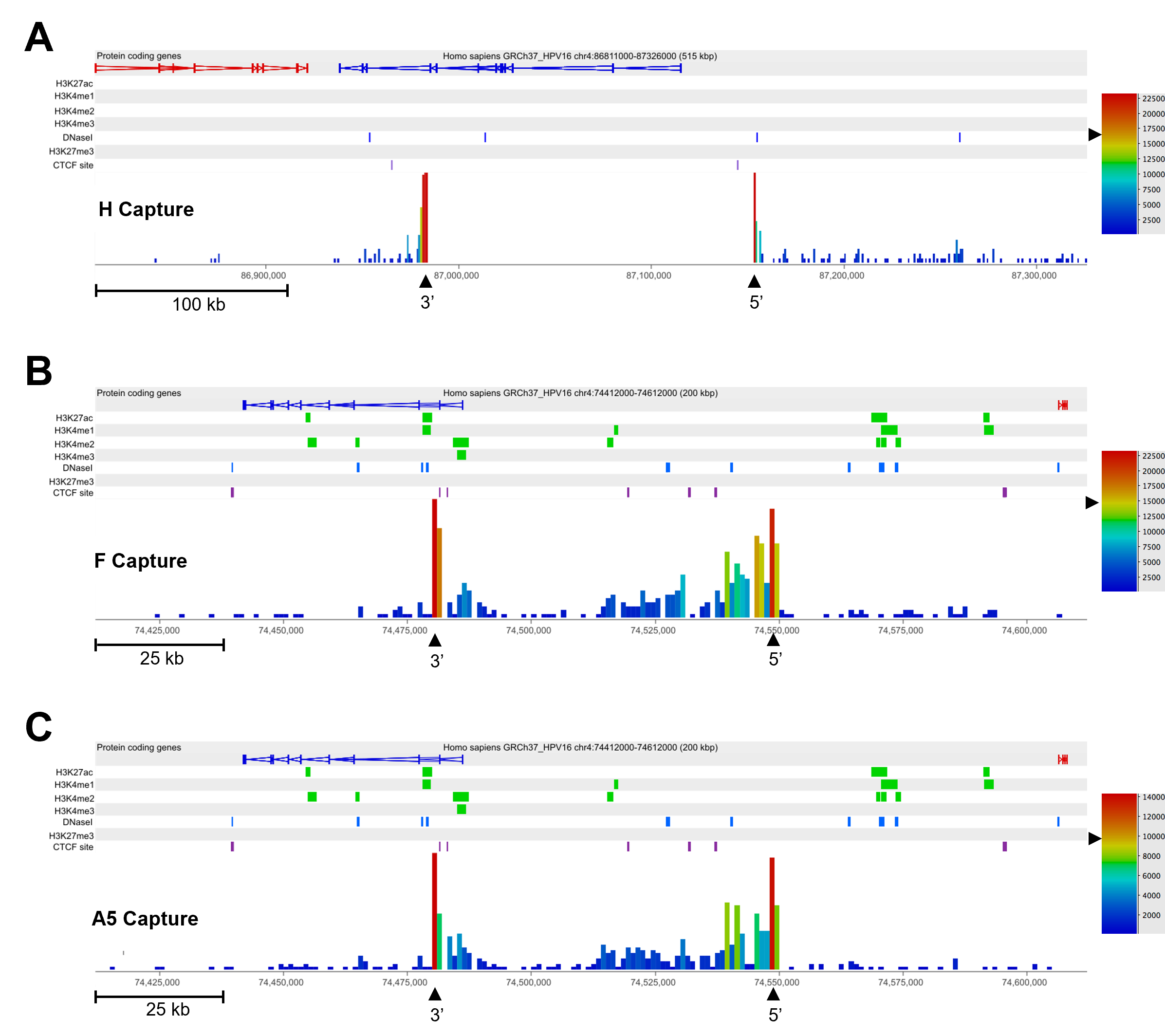

Supplement: S11 Fig — (A) Capture Hi-C data is presented 515 kbp across the HPV16 integration locus for W12 clone H. The 5’ and 3’ breakpoints of the virus are indicated by the tallest red bars and are labelled with black arrowheads, running leftward due to the direction of virus sequence and without intermediate reads due to deletion of host sequence during ‘direct’ integration mechanism. (B) Capture Hi-C data is presented 200 kbp across the HPV16 integration locus for W12 clone F and (C) for W12 clone A5. The 5’ and 3’ breakpoints of the virus are indicated by the tallest red bars and are labelled with black arrowheads, being inverted in comparison to the direction of host sequence due to the ‘looping’ integration mechanism. In each panel, the scale bar represents the normalised read count. Additionally, protein-coding genes are shown in the first track, followed by the alignment of ChIP-seq data from the NHEK cell line (ENCODE). Post-translational histone modifications of host enhancers (H3K27ac, H3K4me1; green), active promoters (H3K4me2, H3K4me3; green), repressed chromatin (H3K27me3, red), DNaseI hypersensitivity sites (blue) and CTCF sites (purple) are shown. Coordinates presented for each window are indicated at the top of each figure. (TIF) [file ppat.1009875.s011.tif]

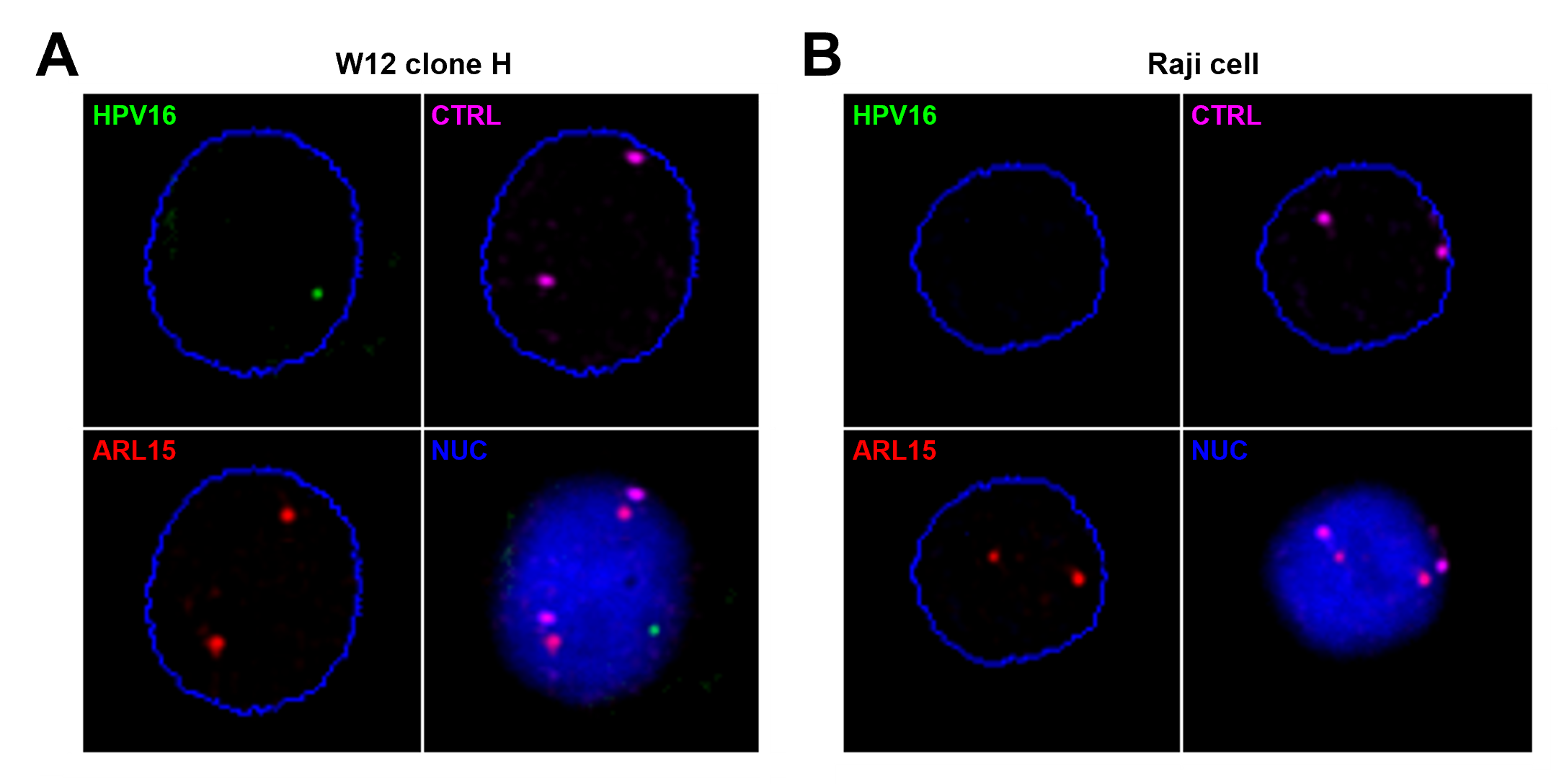

Supplement: S12 Fig — Representative images of probes (used for assessment of W12 clone G2 cells in Fig 4) hybridised to the genome of one (A) W12 clone H cell (n = 1327) and one (B) Raji cell (n = 1284) in a 3D FISH experiment. Probes target: HPV16 (green); Control region (CTRL) (Chr5: 51,676,020–51,873,551; purple); and ARL15 (Chr5: 53,473,886–53,584,235; red). Nuclear boundary is defined as blue circle and a composite image (bottom right) is present with DAPI (NUC, blue) stain. The single HPV16 integration site in W12 clone H is present on chromosome 4 (Chr4: 86,983,196–87,153,458, with host deletion), whereas Raji cells are HPV-negative. (TIF) [file ppat.1009875.s012.tif]

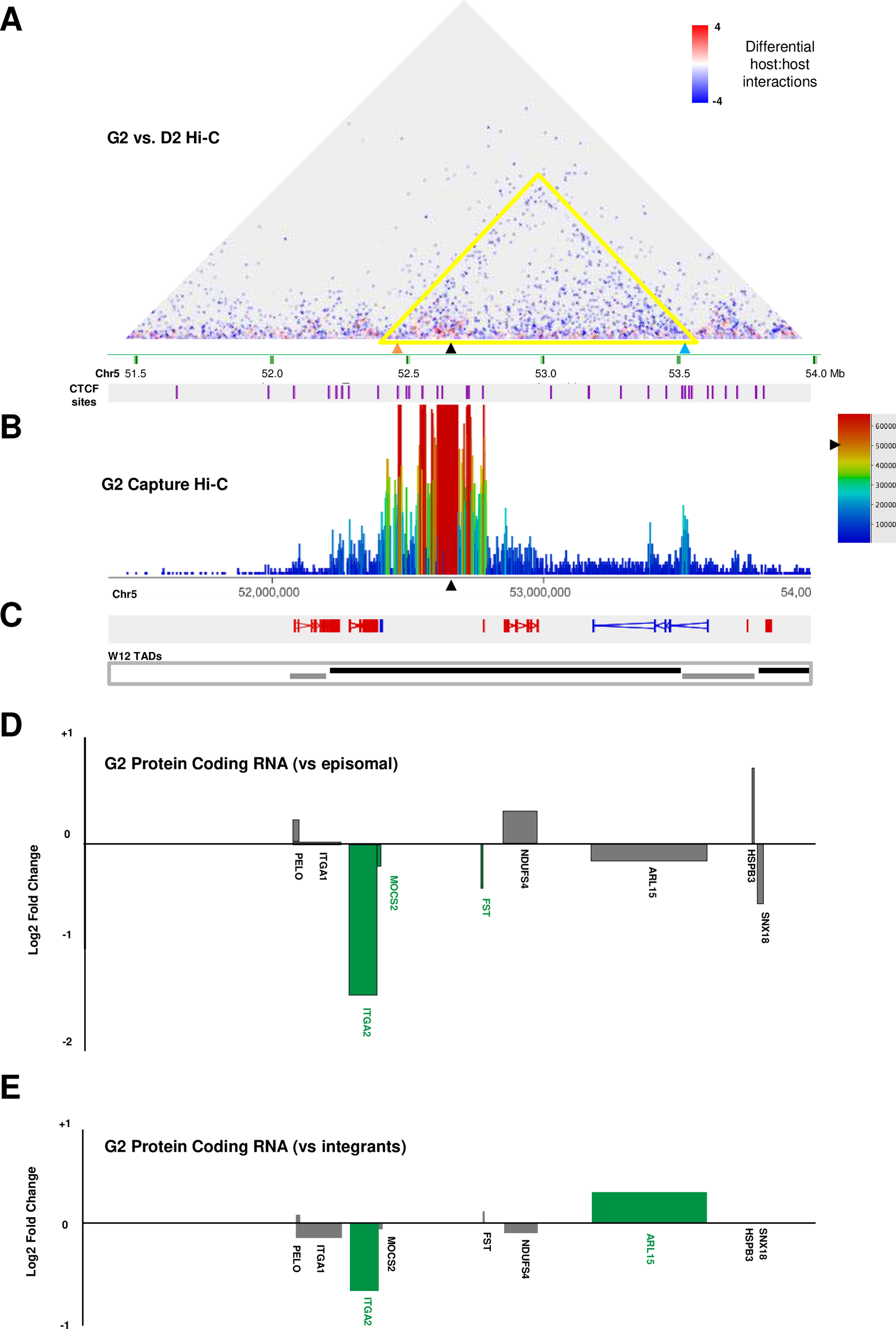

Supplement: S13 Fig — (A) Comparative analysis of Hi-C libraries between clones G2 and D2 shows significant differentially interacting regions (DIRs) within the TAD of integration (red–increased host:host interactions in clone G2 vs D2; blue–decreased host:host interactions in clone G2 vs D2), including the decreased interaction in clone G2 between the ARL15 locus/TAD boundary (Chr5: 53.52Mbp, turquoise arrowhead) and the locus between MOCS2 and FST (Chr5: 52.4Mbp, orange arrowhead), highlighted as a ‘yellow outlined triangle’. HPV16 integration site is indicated with a black arrowhead. Data is aligned to sites of host CTCF interaction (purple lines). (B) Associated Capture Hi-C data is presented across Chr5: 51.5–54 Mbp. HPV16 integration site is indicated with a black arrowhead (scale bar represents the normalised read count). (C) Aligned protein coding genes (rightward, red; leftward, blue) and the extent of W12 topologically associating domains (TADs) are shown below. Charts are presented indicating the transcript level of host protein coding genes within the 2.5 Mb region of W12 clone G2 relative to (D) W12 episomal (Par1) levels and (E) the mean transcript levels of all other integrants used in the study. All data is shown as a Log2 fold change with significant changes (p<0.05) indicated by green bars. Gene length is indicated by width of the corresponding bar. (TIF) [file ppat.1009875.s013.tif]

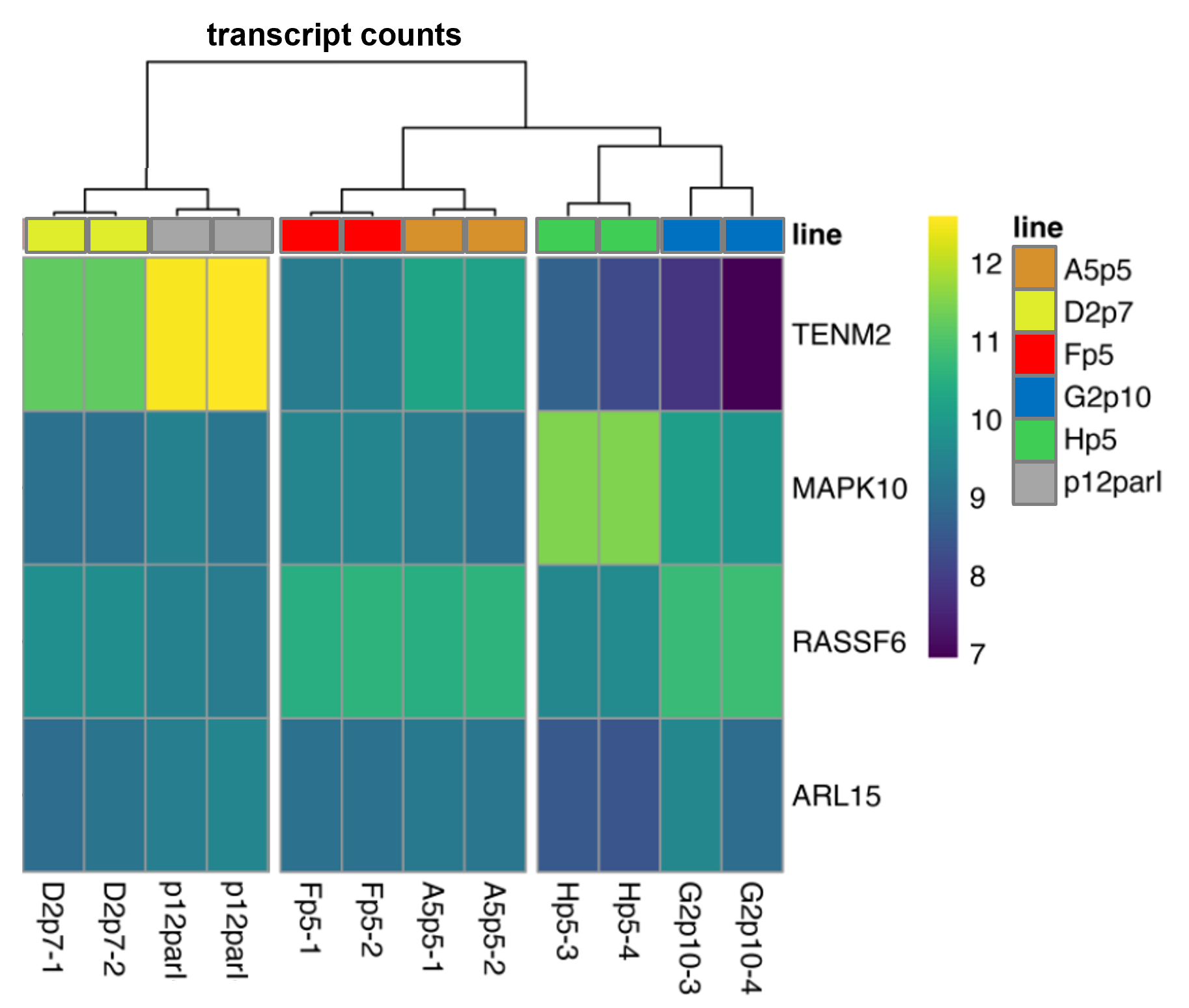

Supplement: S14 Fig — Heatmap of normalised transcript counts from RNA-seq data of the host genes at which HPV16 integration occurred in clones D2 (TENM2), H (MAPK10), F/A5 (RASSF6) and the gene of virus:host interaction in clone G2 (ARL15). (TIF) [file ppat.1009875.s014.tif]

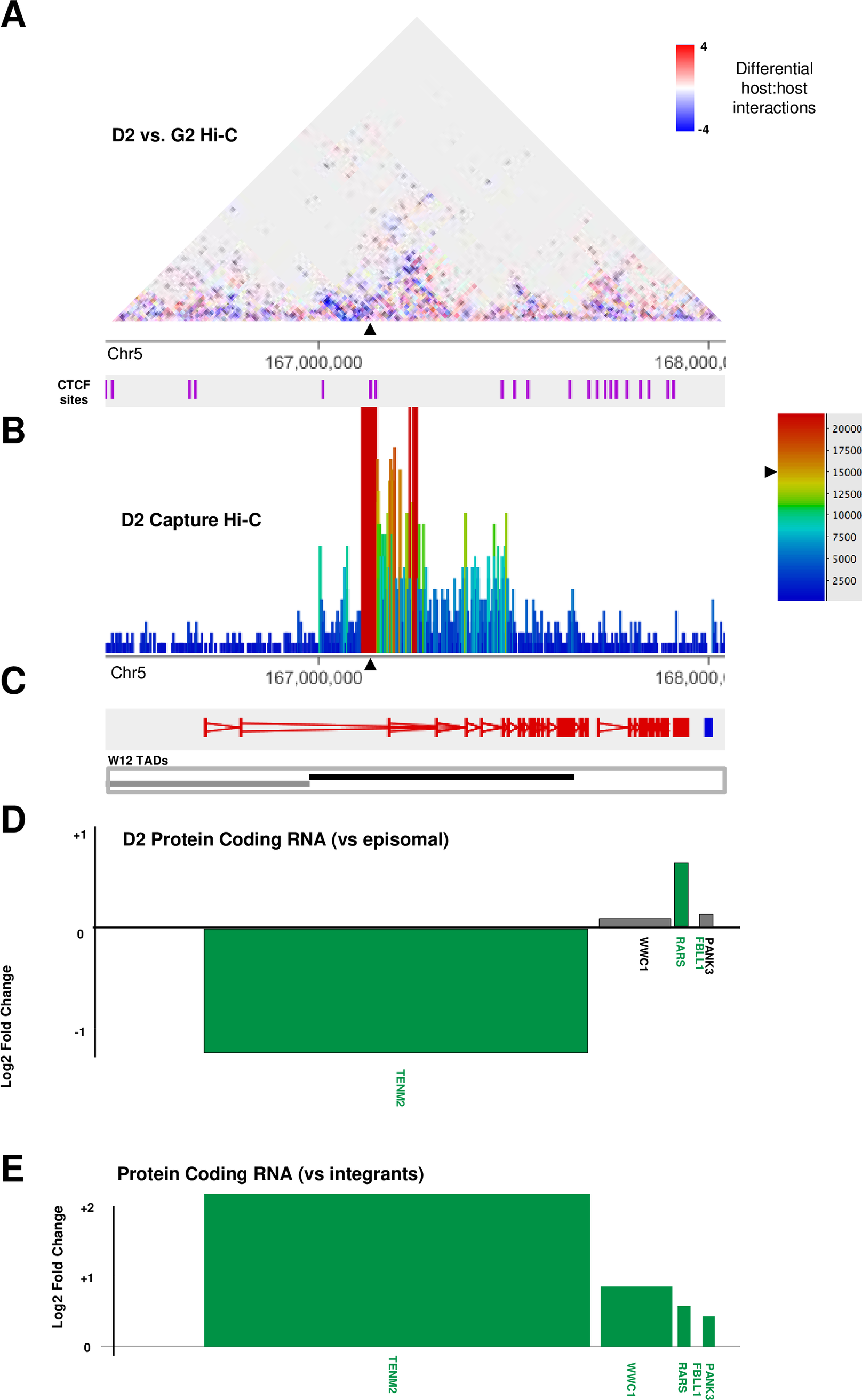

Supplement: S15 Fig — (A) Comparative analysis of Hi-C libraries between clones D2 and G2 shows no significant host:host interaction changes within the TAD of integration (red–increased host:host interactions in clone D2 vs G2; blue–decreased host:host interactions in clone D2 vs G2); aligned to sites of host CTCF interaction (purple lines). HPV16 integration site is indicated with a black arrow. (B) Associated Capture Hi-C data is presented across Chr5: 166.5–168 Mbp. HPV16 integration site is indicated with a black arrow (scale bar represents the normalised read count). (C) Aligned protein coding genes (rightward, red; leftward, blue) and the extent of W12 topologically associating domains (TADs) are shown below. Presented charts indicate the transcript level of host protein coding genes within the 1.5 Mb region of W12 clone D2 relative to (D) W12 episomal (Par1) levels and (E) the mean transcript levels of all other integrants used in the study. All data is shown as a Log2 fold change with significant changes (p<0.05) indicated by green bars. Gene length is indicated by width of the corresponding bar. (TIF) [file ppat.1009875.s015.tif]

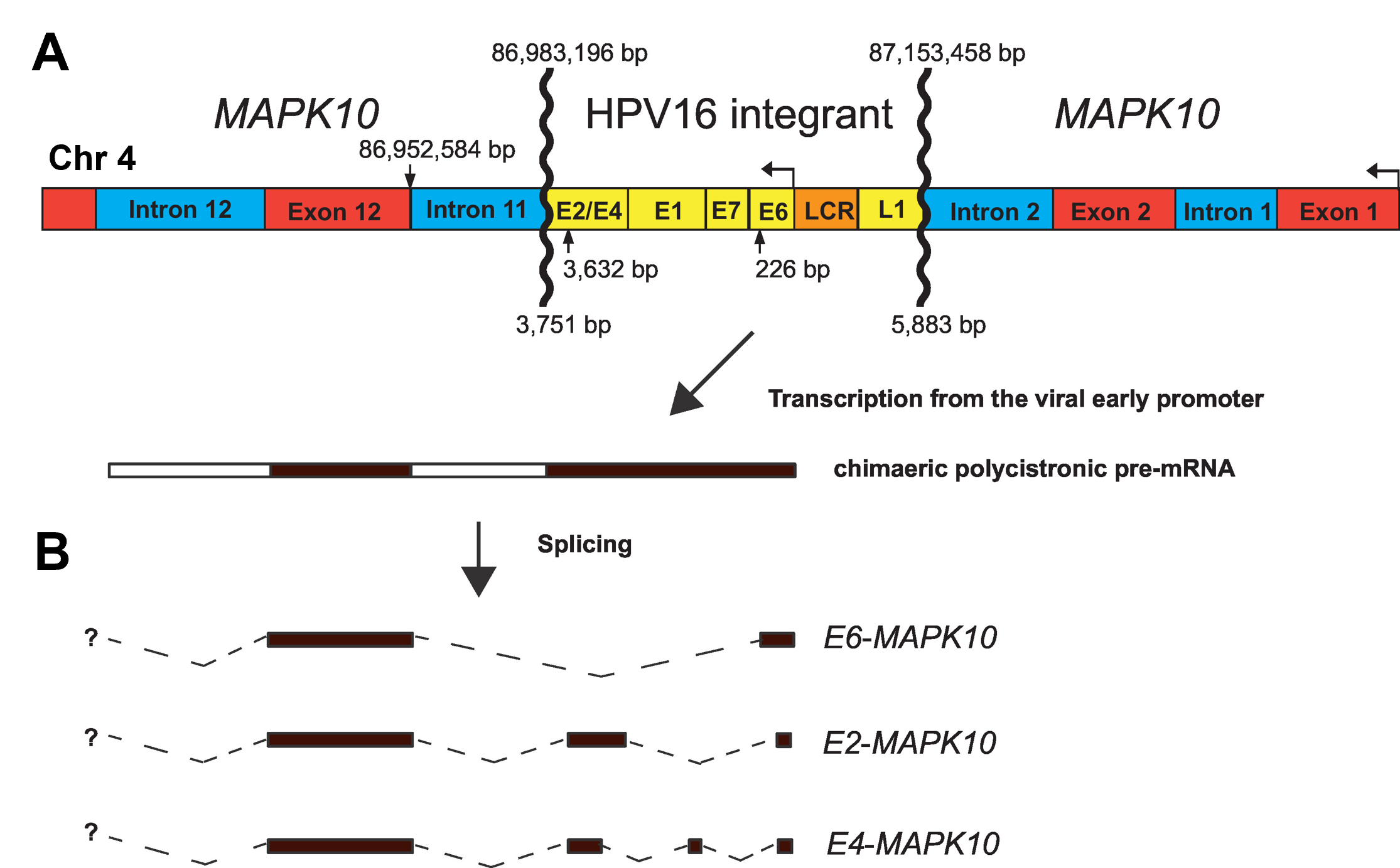

Supplement: S16 Fig — (A) Diagram summarises HPV16:host breakpoints and deletion of introns/exons in MAPK10 gene with (B) spliced fusion transcripts found by RNA-sequencing in W12 clone H. (TIF) [file ppat.1009875.s016.tif]

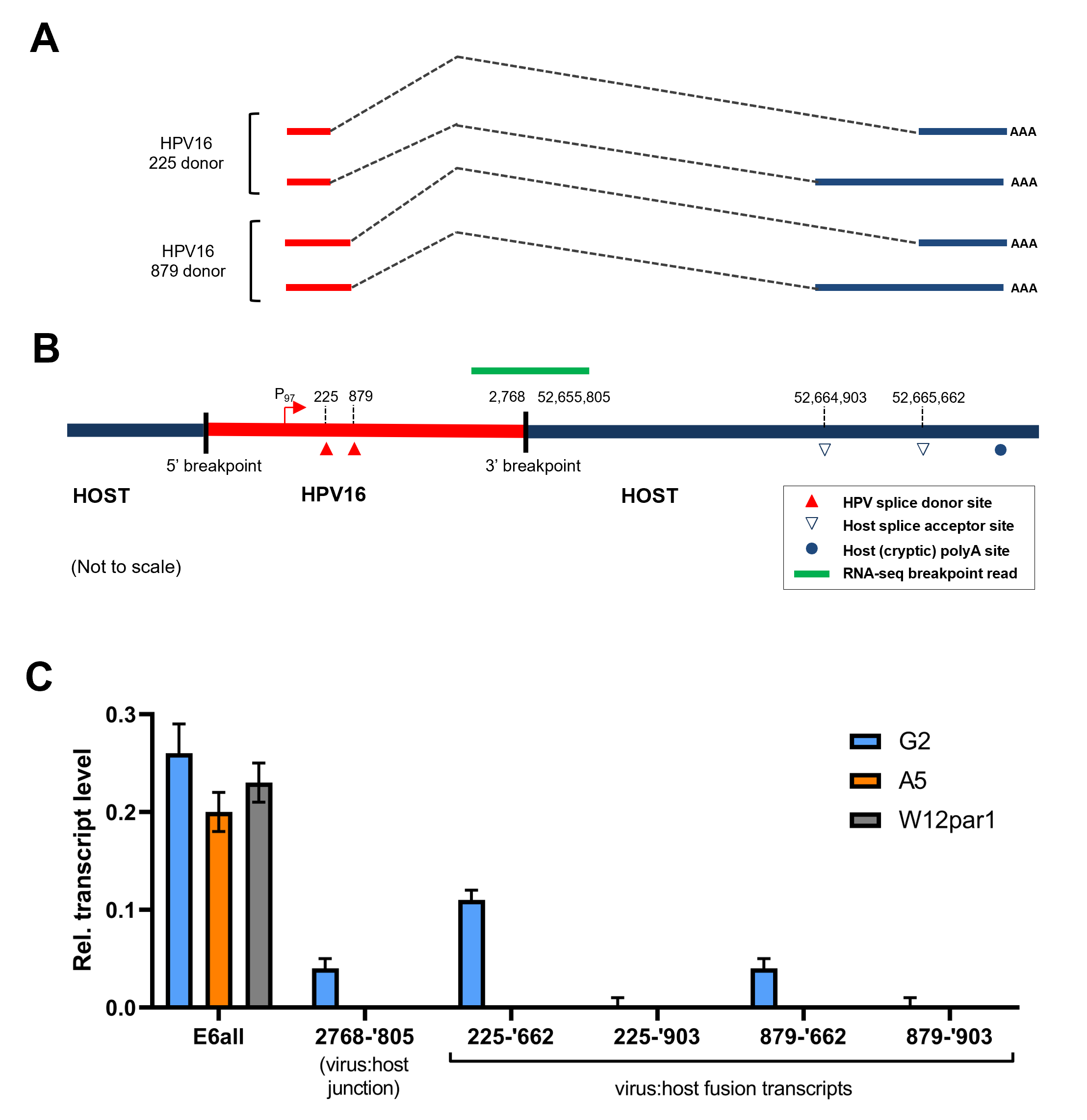

Supplement: S17 Fig — (A) Diagram summarises HPV16:host fusion transcripts found by RNA-sequencing in W12 clone G2. (B) Mapping of the fusion transcript HPV16 splice donor locations and host splice acceptor sites. (C) Determination of G2-specific fusion transcript expression levels via qPCR in comparison to total E6 coding transcripts (E6all) in clones G2 (blue), A5 (orange) and episomal W12par1 (grey). (TIF) [file ppat.1009875.s017.tif]

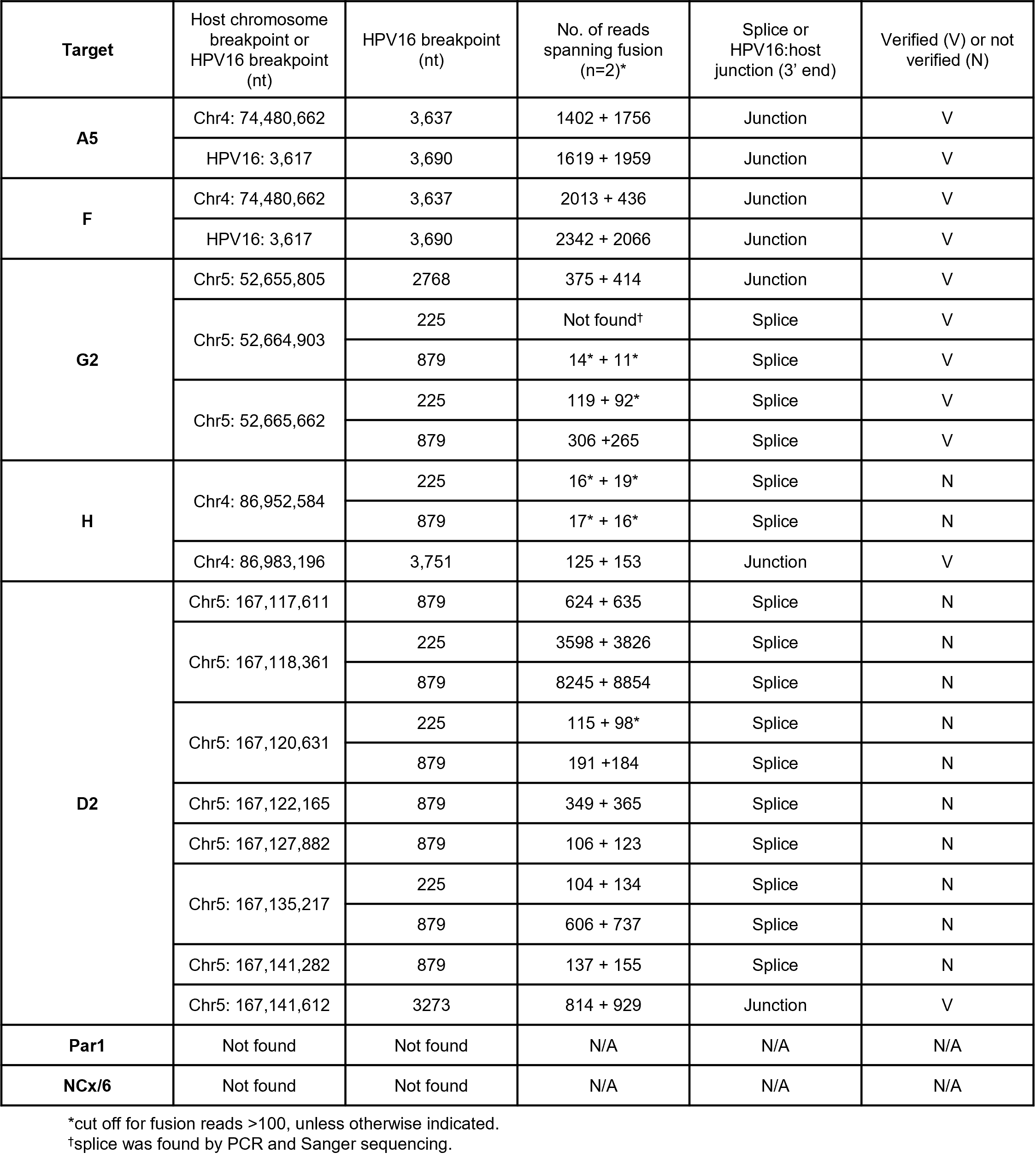

Supplement: S1 Table — (TIF) [file ppat.1009875.s018.tif]

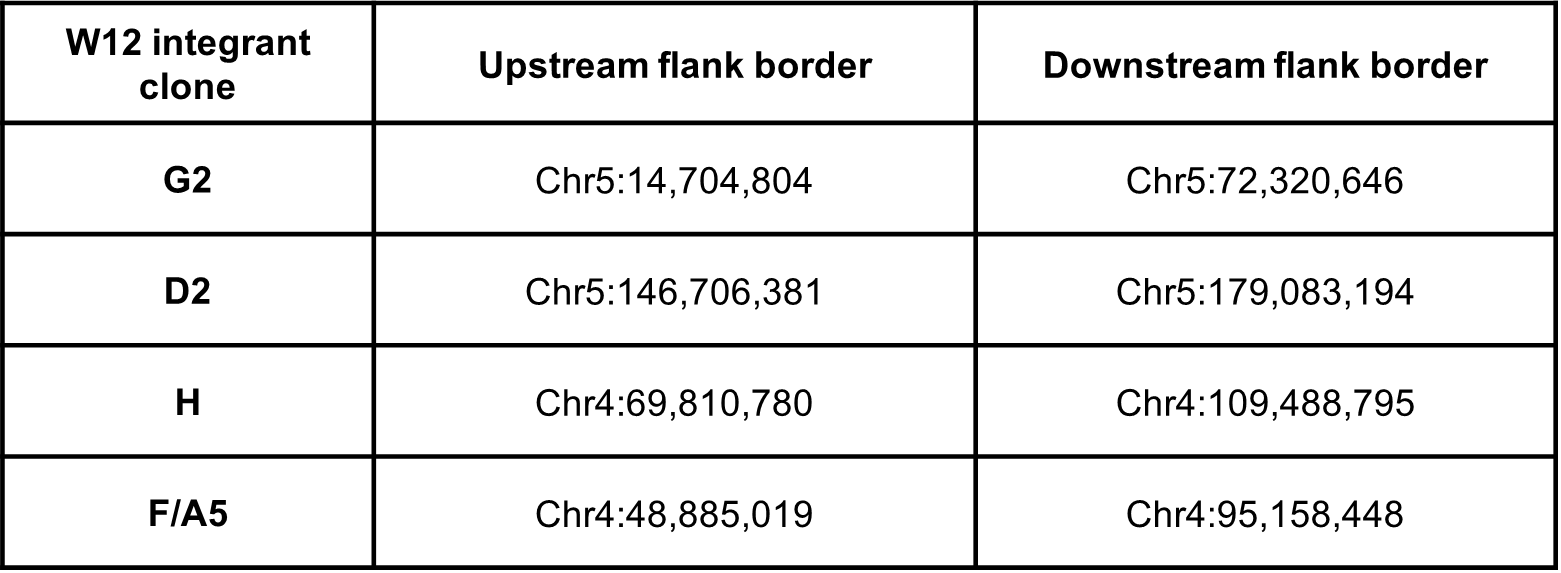

Supplement: S2 Table — (TIF) [file ppat.1009875.s019.tif]

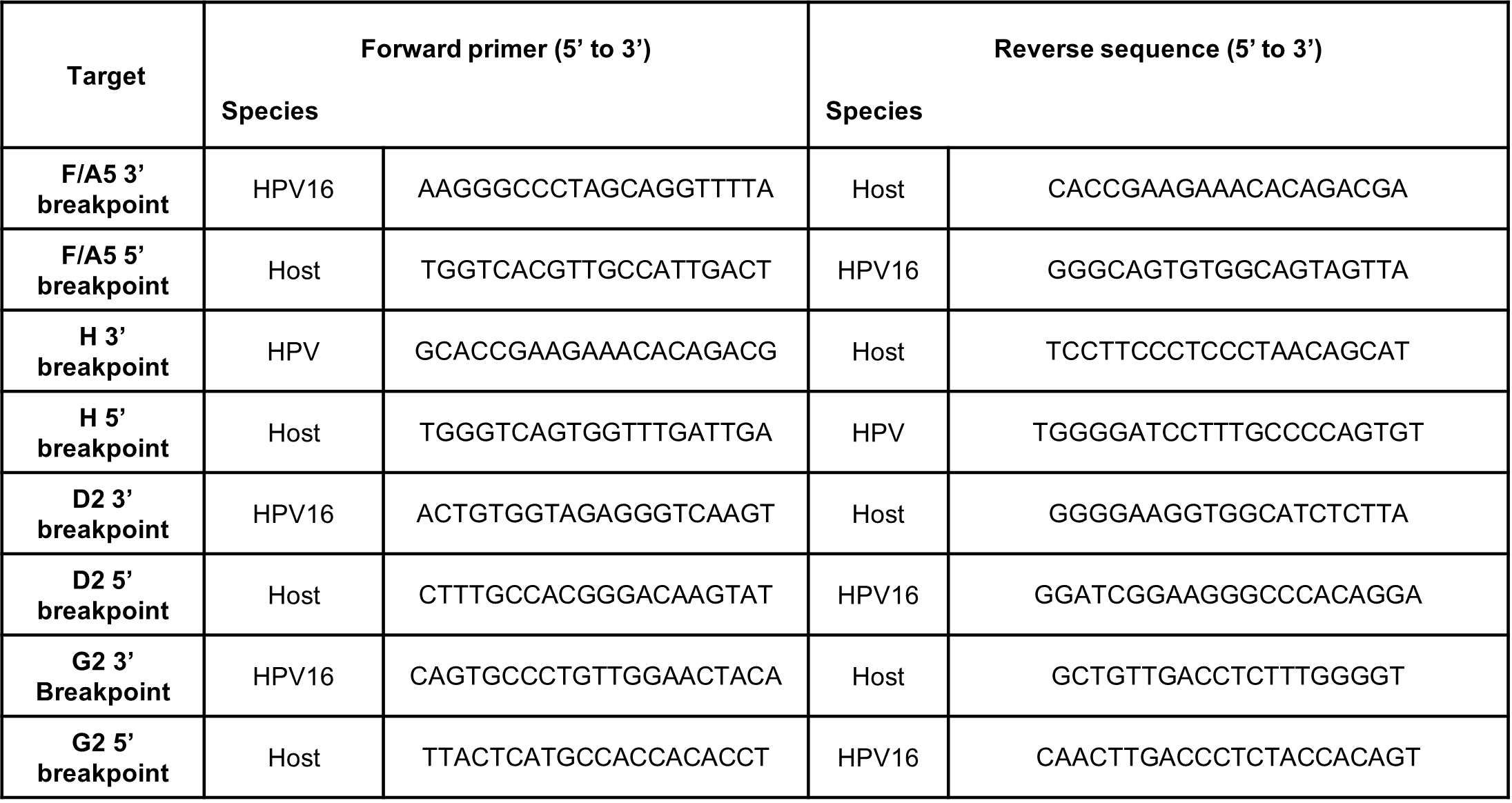

Supplement: S3 Table — (TIF) [file ppat.1009875.s020.tif]

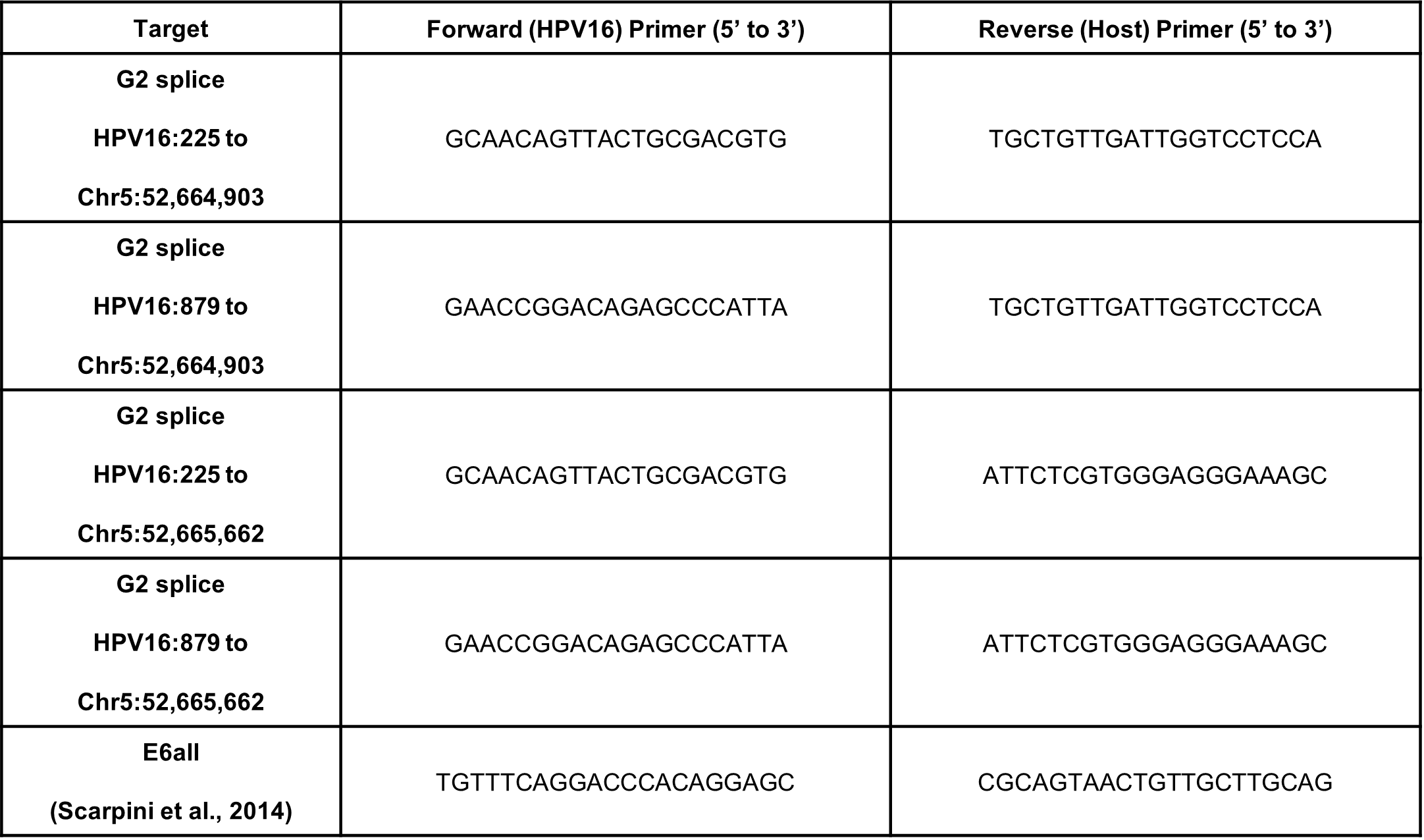

Supplement: S4 Table — (TIF) [file ppat.1009875.s021.tif]

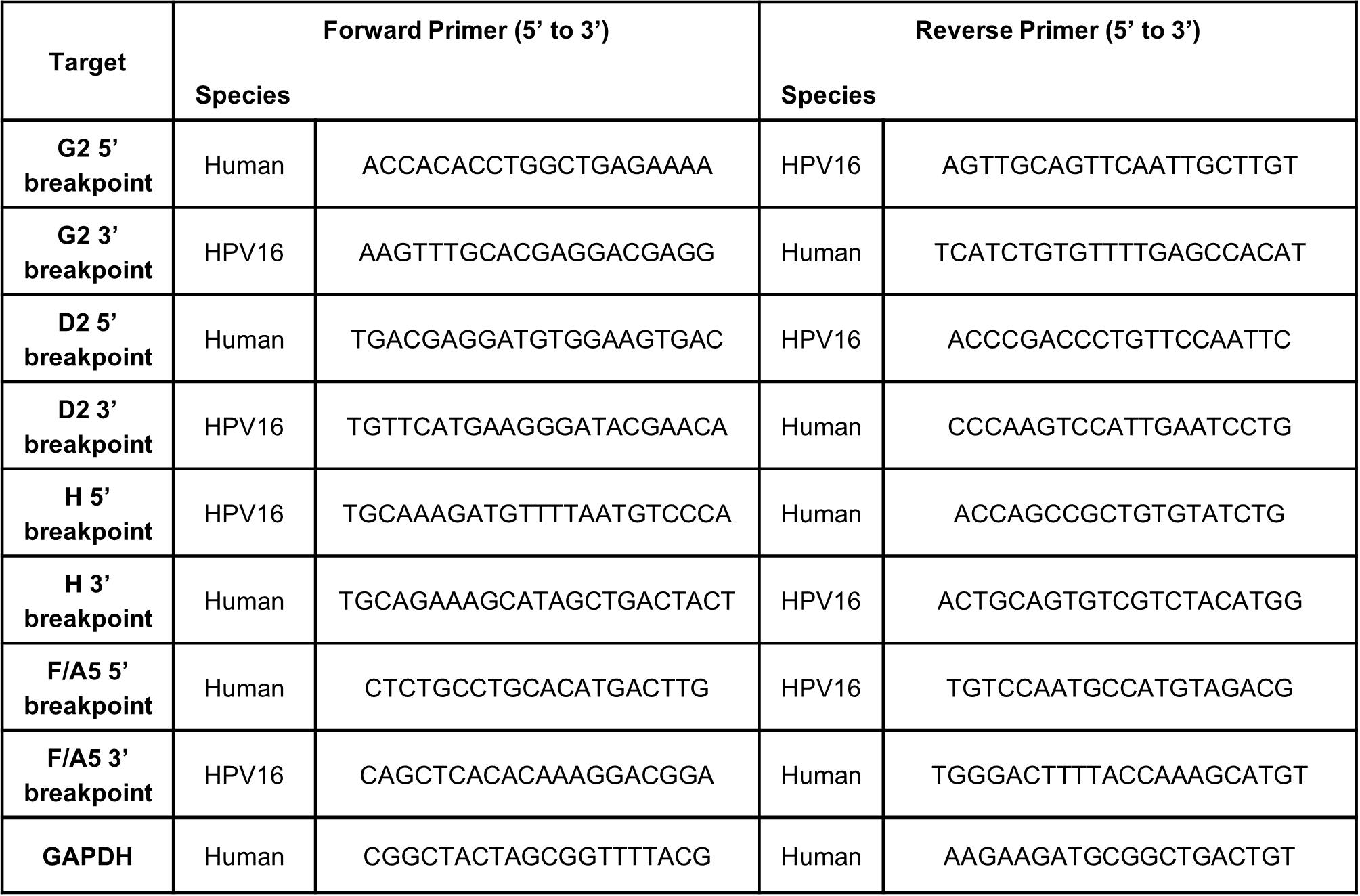

Supplement: S5 Table — (TIF) [file ppat.1009875.s022.tif]

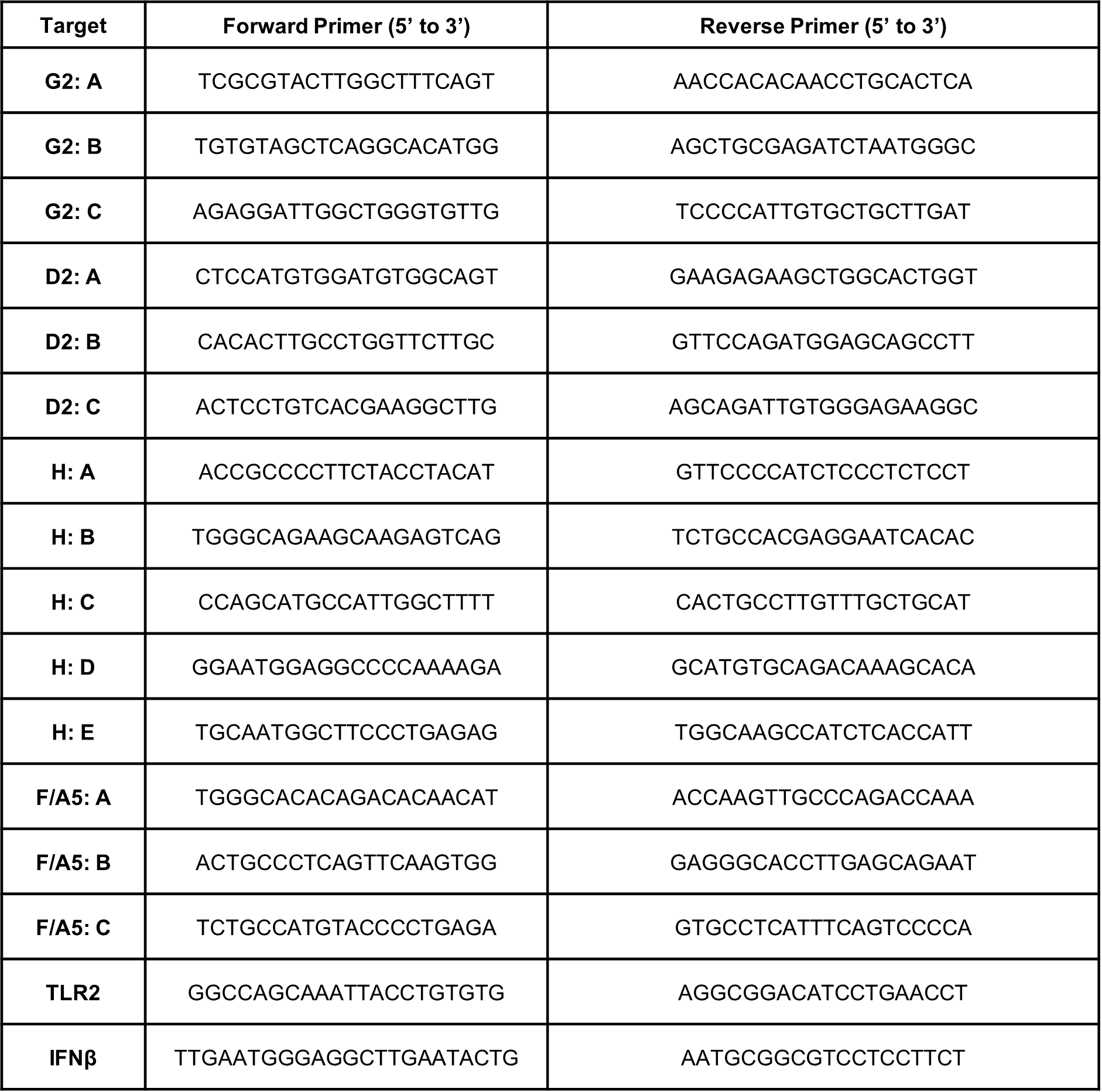

Supplement: S6 Table — (TIF) [file ppat.1009875.s023.tif]

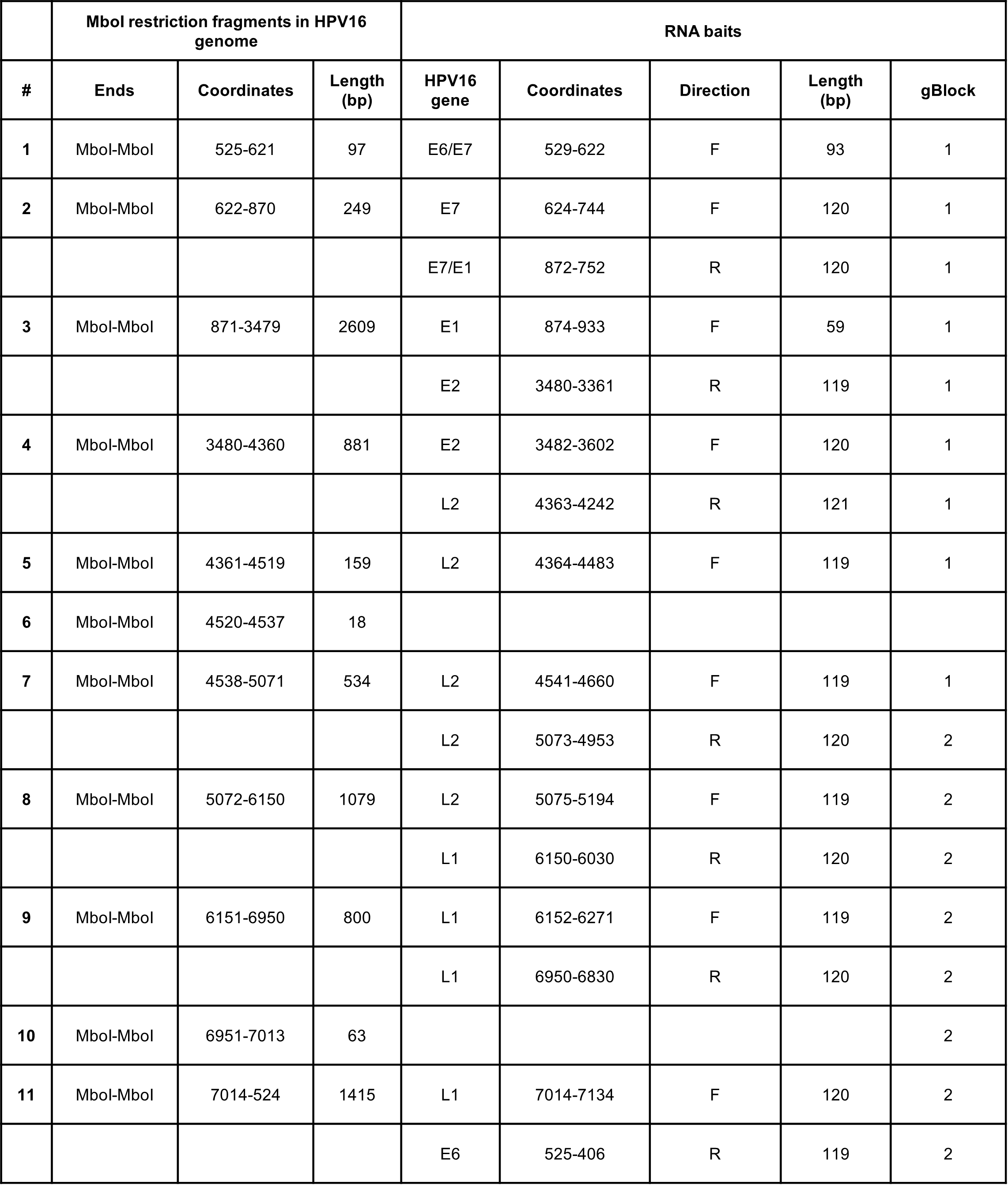

Supplement: S7 Table — (TIF) [file ppat.1009875.s024.tif]
