# Supplementary material for: Short- and long-range cis interactions between integrated HPV genomes and cellular chromatin dysregulate host gene expression in early cervical carcinogenesis
Source: PLoS Pathog. 2021 Aug 25;17(8):e1009875. doi: 10.1371/journal.ppat.1009875 (PMC8439666; doi:10.1371/journal.ppat.1009875)
Supplement: Attachment — Submitted filename: 210805 Response to Reviewers comments.pdf [file ppat.1009875.s026.pdf]

Response to Reviewers report

Reviewer #1

The study by Graves et al. has addressed many of the concerns raised in the initial review. The authors conclusions have been tempered and confusing use of jargon has been clarified. The modified discussion of the significance of effects of integration on TADs and expression of associated genes is useful. The study provides some interesting insights into gene expression from integrated copies of HR-HPVs.

Thank you.

Reviewer #2

(No response)

Thank you.

Reviewer #3:

The strengths are good and weaknesses are addressed below under Minor Issues. The novelty, significance, execution and scholarship are good. The revised manuscript is much clearer.

Thank you for this appraisal. It is much appreciated.

The authors present striking and dramatic results in which HPV DNA that has integrated into the human genome can closely associate in cis with distally located human DNA and modulate gene expression at those locations. It's proposed that the associated distal human DNA is not associated by recombination between the human DNA and HPV DNA, but by non-covalent interactions. Since covalent linkage is the most obvious explanation, the authors must ensure there are no recombination events that explain the association. The authors needed to show that the distal human DNA associated with HPV DNA was is not associated via covalent linkage by showing there are no aberrant junction reads or fragments from within the distal human DNA region that might explain its connection to HPV DNA. The authors state something to this effect in the response to reviewers but I don't see anything in the manuscript so that the readers can know this. It should be clearly stated. Also, the authors should present the odds of not finding a junction should they exist near the distally associated human reads based on the number of reads in the area. This will give the readers confidence in the negative result.

We have now run the DNA sequencing data through 'LUMPY', a probabilistic framework for structural variant discovery (PMID 24970577) (Methods now updated in lines 905-913), and found only the 5' and 3' breakpoints, which is consistent with our previous analysis; hence, no covalent linkages elsewhere. This is now clearly explained in lines 219-223 and 293-297, including the statement that 'with a very high likelihood (binomial test, p<0.00001) that virus:host looping interactions were not aberrantly called due to covalent linkage between HPV16 and host genomes at further breakpoints'.

Box plots in fig 4 are still confusing. If the distribution of values is not normal then non-parametric comparisons make sense, such as box plots shown, but is this true? If the data follows a normal distribution, then the means should be compared. What numbers are presented below the plots? The original manuscript said they were mean ± se but response to reviewers said median and legend now says nothing. If median then what are the ± values? Instead of a fairly standard test for comparing non-normal data, like Mann-Whitney, the authors use the Fisher's exact test. While this reviewer can envision a way to group the values based on and relative to the median, it is not clear how the authors grouped the values for the Fisher's exact test.

Thank you for highlighting this issue and many apologies for the confusion. The statement of use of the Fisher's exact test was erroneous and the legend should have stated (as it does now in lines 1308-1310) that the numbers below the box/whisker plots are 'mean ± SEM' from which an unpaired, two-tailed Students T-test was conducted, consistent with the reviewer's point above. This has now been corrected.

The Fisher's exact test was used for comparing the reads found in the region around the HPV integration site and another region, but it's not clear how the authors did the necessary grouping.

Our explanation of this methodology, including the choice of the regions analysed, has been improved across lines 311-315.

For the F test for variance in bin gene expression in regions in Fig 8, p-values still need correction for multiplicity. Only four p-values in H, F and A5, look significant with this reviewer's corrections using an FDR of 0.05. The FDR needs to be stated.

In Figure 10, multiplicity has now been corrected for using a 'Benjamini Hochberg correction' (as stated with the FDR in the Methods, lines 952-954) and the description of data in Results updated accordingly (lines 449-451).

Line 391: "the mean of several clones lines" is not the comparator but a comparate; the means by which the comparates are compared is the comparator.

Thank you. This has now been corrected to 'comparate', as requested above, in line 400.
