# Supplementary material for: Short- and long-range cis interactions between integrated HPV genomes and cellular chromatin dysregulate host gene expression in early cervical carcinogenesis
Source: PLoS Pathog. 2021 Aug 25;17(8):e1009875. doi: 10.1371/journal.ppat.1009875 (PMC8439666; doi:10.1371/journal.ppat.1009875)
Supplement: Attachment — Submitted filename: 210617 HPV Hi-C rebuttal.docx [file ppat.1009875.s025.docx]

Editor’s feedback:

All three reviewers appreciated strengths of the manuscript and found the study to be of interest and significance, but also pointed out important areas to be addressed in revision. Please pay close attention to the points by each of the reviewers, which will be important to address in your point-for-point rebuttal. The reviewers each point out a number of areas that will be important to address, including the importance of revising the writing in a number of places to improve clarity, soften/qualify conclusions that are speculative, to better showcase new vs old findings, and to better discuss effects of how host genome structural alterations at HPV integration sites might also contribute to local nuclear architecture and gene expression profiles. As pointed out by reviewer #1, important areas for a revision should include re-analysis of the significance of the observed looping between viral and cellular sequences, to address whether 2-fold changes in host gene expression result in biologically significant phenotypes and to thoroughly discuss this point; re-analyzing in Fig. 2b which genes are significantly changed in expression in the areas with high levels of looping. As commented on by reviewer #2, an important control is missing in Fig. 4, and higher resolution images should be provided and 4B should be changed to improve clarity. The Dixon et al. TAD calls should be reprocessed to match their own W12 subclone Hi-C data, and RNAseq analysis should be done to compare profiles of expression changes within TADS/chromosomes relative to that of W12 parental subclone. As pointed out by Reviewer 3, data should be provided to show growth advantage of integrant clones relative to cells with episomal HPV DNA, to provide more evidence that associated human DNA is statistically significantly above background or covalently joined to HPV DNA with mappable junctions. Likewise, evidence of statistical significance should be added to Figs. 2,3, 6-8 and technical issues with the Fig. 4 FISH analysis should be addressed.

*We thank the editor(s) and all reviewers for their extensive review efforts and proposing improvements to the manuscript. Below are the responses to the reviewers’ comments (including those highlighted by the editor(s)) and, as requested, all text changes within the manuscript itself are denoted by red highlighted text.*

Reviewer's Responses to Questions

Part I - Summary

Please use this section to discuss strengths/weaknesses of study, novelty/significance, general execution and scholarship.

Reviewer #1: Groves et al. describe a Hi-C analysis of HPV 16 positive cell lines that contain integrated copies of HPV 16 derived from a parental W12 line derived from a biopsy of a precancerous lesion. The authors conclude that HPV 16 genomes integrated into host chromosomes in regions of open chromatin and short duplications or deletions occur at regions flanking the integrated genomes. Hi-C analysis indicates looping between viral sequences and cellular sequences that appear to primarily localized with in the same TAD that contains the integrated viral genome. Interestingly, integration does not lead to changes in expression of other genes in the TAD greater than 2-fold exclusive of the gene proximal to the integration site . While the studies are of interest, a number sections of the manuscript are not clearly written and need to be improved. The significance of the looping studies needs to be discussed

*The improvements to the text of the manuscript detailed below by Reviewer#1 have now been conducted. Please see below sections for a point-by-point response.*

Reviewer #2: The authors present a novel ‘HPV16-specific Region Capture Hi-C’ technique to dissect viral-host chromatin interactions at HPV integration sites and correlate this with local changes in gene expression profiles. They show that both short and long-range three-dimensional (3D) interactions between the integrated viral genome and host chromosomes can modulate host gene expression changes through the disruption of local host:host 3D interactions known as topologically associating domains (TADs) and suggest this as a potential mechanism for clonal selection of HPV integrants at the early stages of cervical neoplastic progression. Using state-of-the-art techniques, these findings extend on earlier work in HeLa cells that have eluded to long-range interactions at the HPV integration site at the MYC locus and provide valuable insight into the role and mechanism of HPV integration in neoplastic progression. The data presented gives a very detailed analysis of viral-host interactions profiled in several subclones of the HPV16-positive W12 cell line, however, more discussion of the effects of associated host genome amplifications, deletions or rearrangements at the integration locus in the context of local nuclear architecture and gene expression profiles would really benefit this manuscript.

*Further discussion of the effects of associated host genome amplifications, deletions or rearrangements at the integration locus in the context of local nuclear architecture and gene expression profiles has now been included as detailed in the point-by-point rebuttal below.*

Reviewer #3: This paper presents the use of a well-established cervical cancer model (W12) for studying HPV, integration and biological outcomes. The authors isolated clones of integrated HPV, and identified HPV DNA junctions with human DNA. Five clones with HPV-human DNA junctions were isolated, two of which were derived from one parental cell with a recombination event between HPV and human DNA. The authors propose that there is physical association between integrated HPV DNA and human DNA at distal locations on the same chromosome via a looping process, and that genes in the vicinity of the integrated HPV DNA are preferentially deregulated.

There is some confusion with the term “3-D interactions”. This term is used extensively and seems to refer to interactions between DNA of different genomic locations, or between chromatin. Yet, it’s safe to assume that reviewers will regard all interactions within the cell as being 3-D. It’s hard to even think of an interaction that would not be in 3-D. If this term means something more than the obvious then it was not explained sufficiently in the paper.

*We agree with the reviewer that the term ‘3D interactions’ is superfluous and so this has been removed from the manuscript and replaced with ‘genome’ or ‘cis’ interactions, as necessary, to explain the meaning. This has also been changed in the title to ’Short- and long-range* cis*’ interactions.*

It is being proposed that HPV DNA integration and resultant altered gene expression results in a growth advantage over cells with episomal HPV DNA. However, data is not presented as to what growth advantage these integrant clones have over non-integrated cells. These clones could easily not have any growth advantage and thus it could suggest that HPV DNA integration or altered gene expression does not offer a consistent mechanism for growth advantage. This is an important point that seems to be skipped over. It should be noted that there can be substantial clonal variation from a single clone for growth rate and any other homeostatic process, such as gene regulation. Therefore, differences in measurements from the 5 clones isolated may only reflect clonal variation unrelated to HPV integration. What is missing in general is analysis of cells with episomal HPV DNA. It’s difficult to study the effects of HPV integration without studying control cells that don’t have HPV integrated.

*The growth rates of these cloned integrant cell lines have been determined in our previous publication (PMID: 24752734) where we concluded that ‘HPV16 integration in basal cervical cells does not necessarily lead to increased levels of virus oncogenes, or to a competitive growth advantage, when compared with the initiating episome‐containing cells.’ Description of this previous finding has now been added in the introduction (lines 99-101) and discussion has been added in lines 454-456. Regarding the use of W12 episomal cells as a further control; we appreciate the viewpoint of the reviewer, and have added W12 episomal RNA-seq data comparisons to Figures 6-9. However, this is caveated by the fact that isolation of the integrant clones took at least 60 population doublings from the initial parental population (PMID: 18922896), and so given the likely changes to the cells (RNA and DNA) that would occur over this timeframe due to concurrent HPV oncogene expression, we believe that comparison to carefully chosen other integrant clones is a much more appropriate comparison.*

One approach used for studying integrated HPV DNA interacting with nearby human DNA uses chromatin digested into short DNA sequences and crosslinked such that human DNA located far from the HPV DNA will not be isolated with HPV DNA unless it is crosslinked due to non-covalent interaction. The concept that integrated HPV DNA is uniquely associated non-covalently with distal human DNA (hundreds of Kb away) seems to come from a study of HeLa cells. However, HeLa has the HPV18 genome so scrambled that HPV DNA can be found recombined with human DNA throughout the region, and it’s far from clear from that study that distal human DNA is connected to HPV DNA by non-covalent means in addition to covalent means. The possibility the authors in this paper are addressing of specific interaction between HPV DNA/chromatin and nearby human DNA/chromatin is intriguing. However, the evidence the authors’ present to support this possibility is not sufficiently convincing. First, readers would need to see evidence that the associated human DNA is not merely non-specific background DNA that comes through in the purification. Non-random patterns can appear in the non-specific DNA due to PCR/HT sequencing artifacts and due to DNA abundance variation, which is known to happen near HPV integration sites. It would be good to see a representative region of the human genome that’s not near the integration site to assess what a lack of associated DNA looks like. Quantitation of the associated DNA in a region near the integration site being statistically different from all other regions would be required to show that it’s significant. It’s also necessary to rule out the likelihood that the proposed HPV associated human DNA is covalently joined to HPV DNA with mappable juctions. Just sequencing purified HPV DNA and aligning all reads to the human genome to see if this DNA is the same human DNA found in the crosslinked chromatin study would help address this likely scenario.

*Although we agree with the reviewer that some Capture methods do lead to ‘background DNA’ coming ‘through in the purification’, we believe that our data (now with additional statistical evidence described below) does not suffer from this issue. Firstly, the Circos plots (Figure 1), albeit at a host genome-wide scale but high stringency, show very restricted interactions with the integration loci specifically. Next, our ‘zooming out’ in Figures 2 and 3 for clones G2 and D2, respectively, was chosen not just to show the extent to which the virus:host interactions appeared but also, in both cases, the very low level of background reads across a similar, adjacent distance range of genome, as shown on the left side of the integration site in Figures 2C and 3B, as is characteristic of reads associating with the rest of the host genome. In both cases, although reads are occasionally still present, they peak (dark blue bars) at less than 0.01% (clone D2, Fig 3B) and 0.001% (clone G2, Fig 2C) of the highest read peak (red bars) in both windows of view. Additionally, a comparison of read associations with these areas of the host genome between the ‘blank’ and ‘loop’ regions of both Figures 2C and 3B* *by hypergeometric test/Fisher's exact test clearly showed a significant difference (p<0.00001 in both cases) (now added in lines 301-307. Taking into consideration the now added explanation of statistical significance for each interaction determined by the Gothic analysis in Results (lines 182-185) and M&Ms (lines 872-874), statistical analysis of contrast of read distribution across the two areas of both Figures 2C and 3B, and that this analysis methodology has been peer-reviewed and published elsewhere extensively in high impact journals (method reviewed in PMID 30010637), we believe our analysis is entirely adequate.*

*Regarding ruling out that virus:host interactions are not further covalently joined HPV:host breakpoints; the ‘HPV integrated site capture’ (HISC) methodology (overview diagram in Supplementary Figure 1B and data in Supplementary Figures 3-6) was specifically designed to verify our breakpoint findings and indeed at no point were any sites of interaction found using this technique other than those which were directly abutted to the integration sites highlighted by capture-seq methods (Figures 2A, 3A, and supplementary Figure 11). This was previously stated in our initial submission (now lines 211-219) and, overall, we believe sufficient to support that the virus:host interactions are true non-covalent interactions/loops.*

It would be helpful in each section that it be made clear when new general information is being presented and what information confirms what has been found in other published studies. For example, mapping of HPV and human DNA recombination junctions and the affected HPV and human genes has been done before quite a bit, so what does the data in this mapping study tell us that’s different. It might not be different and the mapping is just the needed preparation for the subsequent studies and that could be clearly stated.

*We apologise for the lack of clarity here and have now addressed this in lines 493-497.*

Data is shown how the HPV integration sites and associated human DNA correspond to chromatin markers in the five clones. Figures 2 and 3 do not present any obvious association between the HPV-associated DNA or integration site and the various chromatin markers based on visual interpretation. Others have found a statistically significant association between integration sites and open chromatin and or active genes, as the authors are suggesting in this data, but those other studies analyzed a large number of integration sites to have sufficient power to establish statistical significance. No statistical analysis was done here and the four integration sites in these five clones may not be of sufficient number to reach statistical significance. There’s also a bit of a scientific leap to think that the chromatin marker patterns in the epithelial cells from the ENCODE analysis would look like the pattern within the W12 cells. A study of the patterns in unintegrated W12 versus once HPV had integrated at these sites might have provided new information.

*We thank the reviewer for their important insight. We previously discussed at length the findings of other studies (now in lines 512-526), where we also make note that our integration sites are anecdotally more regularly associated with sites of open chromatin/active chromatin marks of transcription (Supplementary Figure 10), albeit this insight was gained from using NHEK ENCODE data sets. However, the reviewer is correct that the low number of sites studied here due to the focus of the study (i.e. possible virus:host genome interactions) and the extensive methodologies mean that statistical significance could not be reached. This is now noted in lines 515-516. We do believe though that utilising publicly available NHEK ENCODE datasets is sufficient for our analysis due to the robust, multi-target nature of these datasets and also that, although not cervical, normal human epidermal keratinocytes are the next best publicly available dataset of this type. Indeed, other groups have also used this same type of approach in the absence or ability to produce genome-wide ChIP-seq data in their own laboratories to very good effect (PMIDs 29364907, 33023834).*

In Figure 2 and 3, it’s not clear where within the genome map is the integrated HPV DNA in one of more copies. If the HPV genomes occupied space in the map then it would not be possible to align the reference genome with markers and genes. If the HPV genomes were designated by the insertion symbol of a triangle, it was not apparent. All that’s shown is the sites for the two ends of the HPV DNA but no HPV DNA.

*The HPV16 genomes are not present in that mapping as, indeed as the reviewer points out, the host data would not properly align with the window. In both Figure 2A and 3A, the virus:host junctions/breakpoints are indicated by 5’ and 3’ black triangles/arrowheads (i.e. either ends of the amplified region). Due to the scale of the subsequent ‘zoom out’ figures, pinpointing the virus integration site was previously shown simply by where the ‘loops’ originate from. However, we agree that this was not obvious and so both the figure (using inverted black triangles) and the legend have been improved appropriately.*

FISH signals for probes of HPV and distal human DNA were detected in interphase nuclei as a way to assess physical interaction between the DNA locations. There are problems with this analysis. For interphase nuclei, a general rule for being able to resolve two discreet signals (a distance of around 200 nm or higher) of probes less than 40 Kb indicates a genomic linear distance of at least 50 kb between the two probes. The data in Figure 4 indicate inter-probe distances are non-zero and measurable. So any association does not represent physically contact or the values would be zero. Maybe the median included measurements of un-resolved distances between probe signals. Distances in interphase nuclei between probes varies for a variety of reasons and typically is a crude and not definitive measure of how close two probes are in the genome. The presented interphase nuclei probings merely shows that the HPV probe is closer to the ARL15 probe that it is to the control probe. For the integrated site, it looks like the ARL15-HPV probe distance is about half the ARL-control probe distance, which is what the presented genomic map shows. This would represent no physical association between ARL15 and HPV. Other issues are the use of BACs as probes which cover a 100Kb plus distance that will appear not as a spherical signal but an elongated signal or even a squiggle, which makes assessing separation between probes more difficult. Also, use of non-flattened nuclei and 3-D imaging, such as what confocal microscopy offers would allow for more accurate measurements instead of a 3-D nuclei imaged in 2-D.

*We thank the reviewer for their insight and agree that the analysis* ‘shows that the HPV probe is closer to the ARL15 probe tha[n] it is to the control probe’. *Although this point had previously been made (now in lines 315-318), it has now been made clearer in the text that this is not definitive proof of direct interaction of two areas of the genome (‘Therefore, collectively DNA FISH and capture Hi-C data strongly support a direct interaction between the integrated HPV16 genome and the host ARL15 gene in clone G2 (Fig 4B, inset).’ in lines 323-325.*

*Regarding the methodology, the 3D FISH protocols and methods used within this manuscript have previously been peer-reviewed and published as a methodology paper (PMID: 23978815) and also published within several research manuscripts, including within journals PLoS Biology, Nat Commun, EMBO J, Sci Adv, PNAS and Nature (PMID: 17622196, 33828098, 31701553, 31663024, 31451639, 31068695, respectively). Although we appreciate that longer probes may produce an elongated signal, it is clear from our representative images that this is not the case in 100s of cells and, therefore, we believe that this molecular approach and automated method is valid and allows for high-throughput and robust analysis of nuclear localization of up to three chromosomal regions within an individual cell.*

Unfortunately, the FISH image was obscured by the colored plus signs and the actual signals are not visible, so it wasn’t possible to assess the image. It wasn’t clear why the summary of data was shown by box plots and not plotting means and standard errors. It wasn’t clear how the p-values were calculated; non-parametric for box plots or parametric for means?

*As with reviewer #2’s request (below), images are now presented without the computer overlaid crosses for both W12 clone G2 (Fig. 4) and controls W12 clone H (HPV16 is integrated elsewhere in the genome; Chr4) and Raji cells (HPV-negative line) (Supplementary Figure 12), and detailed in lines 320-323. Data was analysed as per previously published methodology (PMID: 23978815) and, due to the range of data, represented by box plots as previously published in associated ‘distance’ studies in PLoS Biology (PMID: 17622196), as now cited in the manuscript. However, in the original version of this manuscript, the explanation of the charts was incorrect; solid line in the box actually denotes the ‘median’. We apologise for this mistake, which has now been corrected in the Figure 4 legend and the explanation of statistical analysis also given (‘Statistical analysis was carried out using a two-sided Fisher exact test’).*

There’s no evidence presented that demonstrates that the HPV DNA and human DNA joined to it is within the human genome as proposed. How do the authors know that the HPV plus ajoined human DNA is not excised and extrachromosomal and replicating autonomously, such as the case with the c-myc gene in cancers unrelated to HPV. A FISH probing of metaphase chromosomes would address this.

*The reviewer’s point that no direct evidence is present within the manuscript to categorically disprove extrachromosomal amplified regions is a valid one (which we previously stated ourselves in (now lines 503-505). However, the collective data we present and that is now discussed in more detail within the manuscript in lines 505-510 strongly argue against this possibility. In short, the 3D FISH data (Figure 4) suggest that the CTRL and ARL15 probes remain within a certain distance of the ARL15/HPV16 signals on the integrated allele (Figure 4C & D), in fact within a similar total range seen between CTRL and ARL15 probes seen on both integrated and unintegrated alleles (Figures 4E & F); this would be difficult to reconcile with a scenario in which the HPV16 genome and flanking regions were dissociated from that chromosome across the nucleus, as is seen with MYC and other oncogene circular extrachromosomal DNA-associated cancers (e.g. PMID: 28178237). Admittedly, our data is 3D interphase FISH rather than metaphase, which is used most frequently. However, we have previously demonstrated using our peer-reviewed/published method (PMID: 23978815) that this technique is sensitive enough to determine dislocation of the HPV16 signal from outlying (outside the amplification region) control regions, which is not seen here.*

For the data in Figures 6 and 7, it would be nice to see that there are flanking regions on both sides of the integration sites in which there’s no gene expression modulation or show a separate representative region with no gene expression modulation. There also needs to be statistical analysis showing if the correlation between integration site and local genes modulated in expression is significant.

*The data in Figures 6 and 7, now including comparison of individual clones to W12 episomal transcript levels (Figs 6C and 7C for clones G2 and D2, respectively) do include short areas of gene transcript levels which, in both cases, are not changed either by fold-change (e.g. clone G2, ESM1-GPX8 locus) or by statistical significance (e.g clone G2, PELO-ITGA1 locus). Additionally, our assessment of host transcript level variance (now Figure 10) clearly shows large regions of genes (albeit in bins of 5) both sides of the integration sites in all clones that are both unchanged at the mean transcript level and certainly not statistically significant (e.g. Fig 10A, bins -10 to 0 and bins 3 to 6). Indeed, this statistically-based variance analysis of whether host gene expression was modulated at, or near to, the integration sites was specifically carried out using the R/Bioconductor package DESeq2 (PMID: 25516281) as this count-based statistical inference tool is able to take account of confounding variables, such as chromosomal/global transcript variance (possibly driven by the HPV oncogenes), and focus on changes in comparison to more precise regions such as our 100 gene flanks. Therefore, given our statistical analysis of individual protein-coding genes in Figures 6 and 7, as well as our more overarching statistical analysis of the broader integration site region in Figure 10, we believe our data and analyses fully support our conclusions..*

For the clones in Figure 7, A5 and F, these are the two clones derived from the same parent cell with the HPV integration event. The gene expression pattern of genes local to the integration site appear to be unrelated. This makes the gene expression analysis for all clones highly suspect. Is the difference in gene expression between the two subclones related to clonal variation?

*We previously stated in the Discussion (now lines 479-482) that ‘More interestingly, we found that clones F and A5 had the same HPV16 integration site, with the same HPV16 breakpoints and inversion, likely indicating that one cell line is a precursor of the other since they exhibit very different phenotypical characteristics’. The inference here relates to our previous publication (PMID: 24752734) where we found that clones A5 and F have differing growth rates and E6/E7 oncoprotein expression levels, which indeed span that of the parental episomal W12 population. We believe that the host RNA-seq data adds further evidence that these two clones have diverged after selection, as the reviewer states is likely due to ‘clonal variation’, and that these cumulative differences (as well as specific differences as highlighted by differential EREG transcript levels in lines 575-578) give rise to differential growth rates. To put more emphasis on this point, we have now added additional text in lines 578-582 in support of this conclusion.*

Figure 8 shows the variance in gene expression as a result of a specific HPV integration event over a 30-50 mb distance covering 200 genes around the integration site with some statistically significant variance yet not for the genes at the site of integration. Why aren’t the genes near the integrated HPV showing statistically significant variance as expected from the gene expression studies in Figure 6 and 7? The clone values are compared to a 6-clone average; what 6 clones would that be? Could the significant variance in gene bins be due to a single gene in each bin? It does not appear that there was any correction for multiplicity in the statistical analysis for the 200 genes. After this reviewer’s correction, none of the significant p-values would appear to be statistically significant.

*Firstly, we apologise for the absence of information regarding the cell lines used and methodology for comparison of RNA-seq data for one clone vs a ‘6-clone average’, and this is now corrected in the M&Ms and throughout the manuscript, appropriately.*

*Regarding the variance analysis; Figures 6 and 7 show individual ‘protein-coding’ genes at the sites of integration having statistically significant change (p<0.05). Previous Figure 8 (now Fig 10), however, shows transcript levels of both ‘protein-coding’ and ‘non-protein-coding’ genes averaged across non-overlapping bins of 5 genes, which may lead to the loss of statistical significance for that bin, hence the lack of significance at the integration site ‘bin’ for all clones but A5. We apologise if this was not clear and this additional explanation has now been added in lines 427-431. Regarding the statistical analysis of variance presented here; we used the R/Bioconductor package DESeq2 (PMID: 25516281) as this count-based statistical inference tool is specifically designed to take account of, amongst other things, outliers such as an individual gene within a bin of 5. Moreover, the system uses an F-test (Figures 10F-J) to determine the most appropriate method of analysis to assess significance due to the variable populations across our sample sets. Due to the robust design of this package, variables such as multiplicity are taken account of and, as such, we feel that the data has been correctly analysed and interpreted fairly.*

What does the term “Mio” used multiple times mean? Million? It should be fixed.

*Apologies, this was a typographical error and has been corrected to ‘million’ throughout.*

Part II – Major Issues: Key Experiments Required for Acceptance

Please use this section to detail the key new experiments or modifications of existing experiments that should be absolutely required to validate study conclusions.

Generally, there should be no more than 3 such required experiments or major modifications for a "Major Revision" recommendation. If more than 3 experiments are necessary to validate the study conclusions, then you are encouraged to recommend "Reject".

Reviewer #1:

1). What is the significance of looping between viral and cellular sequences if only moderate changes in expression of 2-fold cellular genes are observed? A more thorough discussion of this point would be helpful.

*We thank the reviewer for this helpful point and have now added further discussion of our findings at the very early stages of HPV16 integration in the W12 model system in comparison to competitively selected clones or carcinoma tissue in lines 639-644.*

2). Different W12 derived clones exhibit looping between regions of the viral genome and cellular sequences. For Hela cells this looping has been shown to lead to enhanced MYC expression. For the high level looping shown in Figure 2B and C (greater than 25K reads) are there genes like MYC located in those loci that are also significantly enhanced or is expression is it at the 2-fold threshold? In either case it would be useful to describe the genes in these loci whose expression is altered.

*Although no genes with as high an association with cancer as MYC were present within the regions of virus:host interactions found in our study, the change to TENM2 expression (4.79-fold vs other clones) in clone D2 (Figure 3B), for which we do provide strong evidence of long-range virus:host interactions contributing towards, has now been better described (lines 635-639) as well as other affected genes in the G2 integration locus (lines 606-615). Additionally, despite not being associated with virus:host interactions, the differential expression of EREG in clones F and A5 (a possible reason why the two clones have very different growth rate) is also described in lines 575-578.*

3). Many of the reads in Hi-C data that are below 500 are unlikely to be significant and only those that are significantly higher are more likely to be physiologically significant.

*All Hi-C data was processed using HiCUP, which removes invalid and artefactual paired end di-tags by overlaying the di-tags on an in silico restriction digest of the reference. The resulting BAM files were then visualised using SeqMonk (Figures 2, 3, 6-9), due to the user-friendly interface, whilst Gothic (PMID: 28379994) was used to determine statistically significant interactions by a cumulative binomial test; results were filtered on their adjusted p-value (the q-value), where the threshold in this case was set at <0.05. As such, q-values ranged anywhere between 0.049 to 3.043e-13. Thus, our determination of interaction significance was achieved statistically and presented, firstly on a genome-wide scale using Circos plots (Figure 1), before being displayed as ‘loops’ on the associated SeqMonk figures. Whether these interactions appear to have physiological significance was determined by the modulation of host transcript levels from the associated genes (Figures 6 and 7).*

4). Sequencing of HPV integration sites has been described in many previous studies. Have microhomology regions at junctions of viral and cellular sequences been previously described?

*We previously stated (now in lines 482-484) in the discussion ‘Our data also support the growing understanding that integration of HPV genomes takes place, at least to some degree, through microhomology-mediated repair (MHMR) of DNA breaks(25, 26, 29, 32, 40, 42-44)’. However, we have now added a recent report (PMID: 31591214) providing a mechanistic role for HPV16 E7 (lines 487-490).*

5). Line 303: the authors state there are not consensus CTCF sites present in these loci, but looping could also be due to non-consensus sites

*Thank you for the insight. Words to this effect as well as appropriate references have been added to the Discussion in lines 536-537.*

6). Line 339: what is meant by no change in nuclear architecture? What parameters are used and how is this quantified? What is an insulation score?

*An ‘insulation score’ is* *a bioinformatics method to detect enrichment of contacts in Hi-C datasets between downstream and upstream 300kb regions across a particular window of interest. A more positive insulation score indicates a stronger TAD boundary. Quantification occurs by standardized -log enrichment (-log (a/(a+b1+b2)) where ‘a’ is the number of contacts between, and ‘b1’ and ‘b2’ the number of contacts within the upstream and downstream 300kb regions. We used this methodology in our study to determine if HPV16 integration and/or resulting virus:host interactions were affecting the nuclear architecture at the hierarchical level of TADs, which we found did not occur (Figure 5). To address the meaning of ‘no change in nuclear architecture’ in the text, the following changes have been made:*

*Lines 346-347 now changed to ‘did not appear to be affecting the local nuclear architecture at the hierarchical level of TADs* *and their boundaries.’*

*Lines 351-352 now changed to ‘did not appear to affect TAD positioning’.*

7), pg. 5 the statement that the integrated clones generated in this tissue culture model are representative of what occurs in vivo progression to cancer is highly speculative and should be identified as such. Have these integrated clones been derived following interferon selection?

*This statement has been removed and a briefer description of the W12 system moved to the M&Ms section as per reviewer #2’s request. Regarding selection of clones, this was done by limiting dilution under non-competitive conditions, i.e. E2 was retained, such that integrants would be present, regardless of whether or not they had a selective growth advantage after episome loss (PMID: 18922896). This was in contrast to our earlier study where interferon treatment was used to clear episomes, but such a competitive environment leads to the integration event(s) conferring the strongest growth advantage coming to dominate the population (PMID: 18922896). Hence, the clones in our study represent HPV16 integrant events that would more likely represent those that occur naturally after HPV16 infection. A much briefer summary (as per reviewer #2’s request) is now present in lines 660-667.*

8). Line 405: too strong a conclusion

*The wording of this sentence (lines 441-444) has now been moderated to ‘Hence, our findings suggest that integration of HPV16 genomes into host chromosomes could have direct modulatory effects on host gene expression beyond both the immediate locus of integration* *and the indirect effects of virus oncogene expression to average chromosomal expression.’*

9). Is there any looping within a single integrated viral genome such as the CTCF site in E2 with the YY1 site as described previously?

*Thank you for this question. Unfortunately, due to the methodological setup of the sample preparation (including the necessary bait length for the E1 gene region), resolution (1kb) and subsequent sequencing depth, this proved impossible with our libraries. However, the point regarding the E1 bait is now further described in the Results section (lines 185-187) and the legend for Figure 1. Also, we have briefly noted that the above intra-HPV analysis was unsuccessful in lines 472-474.*

9). A number of sections are poorly written with lots of jargon making it hard to understand and should be made clearer.

a). 289-294

b). 335-337

c). 351-352

d). 365-369

*a) This section has now been re-worded, with additional information to aid explanation (lines 278-284).*

*b) The paragraph has been re-written with further explanation of Insulation score analysis (now lines 329-347).*

*c) This section has now been improved with comparison between TAD calling and insulation score analysis added (lines 353-359).*

*d) This section has now been improved with further explanation (lines 376-382).*

Reviewer #2:

Point 1: For Figure 4, as a control, the authors should include FISH analysis in a HPV-negative cell line to control for HPV16 DNA probe background signal and also repeat in a different W12 subclone to show that the interaction between the integrated HPV16 DNA and ARL15 gene is specific to clone G2. A higher resolution image of the FISH signal should be used in Figure 4B. The coloured “+” symbols indicating the probes for the integrated verses non-integrated alleles are covering the FISH signal and should either be removed and replaced with arrows marking the integrated/non-integrated alleles or made smaller and positioned adjacent to the FISH signal

*As with reviewer #3’s request (above), the highest resolution representative images from the automated methodology are now presented without the computer overlaid crosses for W12 clone G2 (Fig. 4). Additionally, data for W12 clone H is now presented clearly showing no local association of HPV16 with ARL15 (HPV16 is integrated elsewhere in the genome; Chr4) as well as data for Raji cells, which are HPV-negative and clearly show no background HPV signal (Supplementary Figure 12) and is described in lines 320-325.*

Point 2: It is stated in the Methods section that Hi-C was performed for all the W12 subclones. For Figure 6, in addition to TADs profiled in Dixon et al., TADs should be called in all W12 subclones for alignment with integration breakpoints in the matched samples to determine whether any of the integration sites disrupt TAD boundaries and consequently gene expression profiles across neighbouring TADs. The authors should reprocess the Dixon et al. TAD calls to match their own Hi-C data in the W12 subclones so that the same parameters for calling TADs across these different cell lines are used for comparison.

*Although Hi-C replicates were prepared for all clones (as stated in the M&Ms), sequencing of the libraries was only conducted on the clones that had statistically significant virus:host interactions beyond that of the immediate virus:host breakpoints and which we believed controlled for one another due to the integration sites, both being on separate regions of chromosome 5, ~115Mb apart. Hence, although we previously inferred that (only) clones D2 and G2 had their Hi-C libraries sequenced (now lines 331-333), we have now made this point clearer in the text (lines 838-841). Indeed, this is why Hi-C profiles and insulation scores were only presented for these two clones. We now present consensus W12 TADs on Figures 6-9 and S13 and S15 as analysis showed no changes to TAD profiles at either site of integration in either clone (G2 or D2) (as newly described in the M&Ms in lines 900-904). Although now outside the remit of this study, determining the TADs and possible changes in further W12 cloned lines at points of HPV16 integration would be beneficial and this has now been stated in lines 587-589. Additionally, the ‘Dixon et al TADs’ have now been removed from the figures due to them having very little relevance to this study (being determined from the normal lung fibroblast cell line, IMR90) and our actual question of whether HPV16 integration, not infection per se, affects TAD boundaries, which in our analyses does not appear to occur in the early stages of W12 progression post-integration (Figures 5, S13 and S15).*

Point 3: For analysis of gene expression changes within TADs and across each chromosome, comparison to the RNA-seq profile in the W12 parental (Par1) subclone that maintains extrachromosomal genomes should be included

*We agree with the reviewer that comparison to parental episomal W12 (Par1) gives an added dimension to the RNA-seq analysis and, as such, has been added here in Figures 6B, 7B, 8B, 9B and D. The findings are then discussed appropriately throughout the manuscript in comparison to the pre-existing analysis versus the mean transcript level of all other clones used in the study. Importantly though, the use of early episomal populations is caveated by the fact that isolation of the integrant clones took at least 60 population doublings from the initial parental population (PMID: 18922896), and so given the likely changes to the cells (RNA and DNA) that would occur over this timeframe through HPV oncogene expression, we believe that comparison to carefully chosen other integrant clones is a much more appropriate comparison. Words to this effect have now been added in lines 390-393.*

Reviewer #3: (No Response)

*Thank you.*

Part III – Minor Issues: Editorial and Data Presentation Modifications

Please use this section for editorial suggestions as well as relatively minor modifications of existing data that would enhance clarity.

Reviewer #1: (No Response)

*Thank you.*

Reviewer #2:

The introduction is very detailed and should be condensed. For example, how the W12 subclones that were used in this study were generated should be briefly described in the methods section and the discussion of the MYC locus should be moved into the final paragraph of the discussion section

*The detailed description of the generation of the W12 integrant cloned cell lines has now been removed from the introduction and is briefly described at the beginning of the methods section. Discussion of the MYC locus has now been moved into the final paragraph of the discussion section.*

Line 153 of the introduction; authors should define type I integrants

*This has now been done: ‘(i.e. retain HPV E6/E7 genes but with deletion or disruption of E2)’ inserted in lines 150-151.*

Line 171 of the introduction; describe what is meant by ‘partially homozygously’ deleted

*This has now been done: ‘with a portion of both alleles within the host break points being deleted’ inserted in line 161.*

Indicate on the Circo plots in Figure 1 the deleted regions within the viral genome for each W12 subclone

*The deleted regions are now denoted in Figure 1 by a shaded/transparent grey box over the region of deletion for each cell line, which is explained in the figure legend.*

Discuss the differences in the distribution of viral-host reads across the HPV genomes for the different W12 subclones and any effects on viral and host gene expression profiles. For example, why is the E1 gene devoid of reads in most subclones? Is that where the integration breakpoint occurred in these subclones? This point is later clarified in the discussion section – also include in the legend for Figure 1.

*Thank you for this direction. The reason for the absence of interacting reads between either deleted regions of the HPV16 genome and/or the E1 gene specifically (due to bait length) has now been added to the Figure 1 legend and to lines 185-187 of the Results section. Additionally, the correlation between integrant virus gene expression profile data (previously published in PMID: 24752734) and the presence of associated interactions with virus genes in this data set is now highlighted in lines 466-469.*

Lines 211-213 of the results section; the authors found that W12 clones F and A5 had the same integration site, with virus-host reads converging to the same region of chromosome 4. How do the transcriptional (viral and host) and epigenetic profiles of these clones compare?

*As with reviewer #3’s request for further discussion on the possible reasons for differences seen between these two identically integrated cloned cell lines, we have now added further text in lines 454-456 that highlights the previous study of virus transcription and epigenetic profiles in comparison to the parental episomal cell line and, indeed, other clones (PMIDs 24752734, 26876196) and also the differences in host transcription profiles found here (lines 573-583), likely due to one clone being a precursor of the other.*

Supplementary Figures 3-5C and 6D are difficult to interpret without having the matched DNA-seq data to visualize where the amplified regions are occurring within the host genome relative to the integrated viral sequences and this should be addressed in the figure. It is not clear what the red arrows are indicating in terms of how the viral genome was co-amplified along with the host sequence. The authors should combine data from Supplementary Figure 7 to better illustrate the architecture of each integration site

*We fully appreciate the reviewer’s perspective here. Unfortunately, however, the study did not involve sequencing of DNA libraries other than for Hi-C, region capture Hi-C and HISC. Therefore, we do not have total coverage of the host genomes but only those portions involved in ‘interactions’. However, we believe that the data provided, including high quality RNA-seq, allow full determination of the virus-host breakpoints (and directionality) in every clone presented and that qPCR suitably determines the copy number of the host regions found within breakpoints by capture-seq. Respectfully, with the issue regarding interpretation of the diagrams as presented in the supplementary figures not having been raised by either of the other two reviewers, nor the editors, and our belief that adding further nucleotide level information from Supplementary Fig 7 could make their presentation less clear (and less comparable across all precise integration data presented together here), we have opted to only improve the figure legends to further aid the reader.*

Lines 327-340; The authors show that the Hi-C heatmaps between clones D2 and G2 are similar across a 5 Mb window, suggesting that integration of the HPV16 genome does not affect the local genome architecture in these two subclones. The 5’ and 3’ integration breakpoints in both subclones are relatively close together (~25 Kb) and do not appear to disrupt any TAD boundaries. Furthermore, there are no complex rearrangements at these integration sites and so disruptions to local genome architecture might not be apparent at this 5 Mb scale. It is possible that integration sites that expand across a large genomic region (e.g. the 170 Kb deletion in clone H), display complex rearrangements and/or disrupt a TAD boundary would affect the local chromatin architecture. The authors should discuss this in the text and consider rewording the title of this results section as not to overstate this finding as this result was only confirmed in two subclones that have a relatively simple integration profile

*We agree with the reviewer that these points had not effectively been discussed and so additional text has been added to lines 595-605, accordingly. Additionally, the title of the results section has been amended to: ‘HPV16 genome integration sites with short-range (~25kbp) host genome amplification do not affect host topologically associating domain boundaries’.*

Lines 346-347 and Figure 6; state which cell lines were used for alignment of integration breakpoints with TADs

*This has now been amended to include a description of the W12 TAD alignment in line 353-357.*

Line 360; make it clear what is meant by “with no restriction to TADs”

*This has been amended to read ‘with no individual direction of change (i.e. up-regulation or down-regulation) of differential expression restricted to individual TADs’ in lines 368-369.*

Lines 365-369; The authors state “Compellingly, upon comparative analysis of Hi-C libraries between clones G2 and D2, a decrease in a host:host interaction was found within the TAD of integration, aligned with the HPV16:host interaction with the ARL15 gene. Hence, changes of gene expression within TADs are possibly due to modulated host:host interactions at this level.” Does this finding not contradict the conclusions presented in results section: HPV16 genome integration does not affect local host genome architecture (lines 326-340)?

*We agree with the reviewer that the initial version of the text easily led to misinterpretation of the findings. This was largely due to the lack of explanation of insulation score methodology (an overall analysis of the region and TAD boundary calling) versus the direct comparison of Hi-C interaction reads (i.e. loops) in Supplementary 13A. This has now been corrected by further describing the insulation score analysis and improving the text both in lines 333-338 and 346-347. The section title has also been amended as such ‘HPV16 genome integration sites with short-range (~25kbp) host genome amplification do not affect host topologically associating domain boundaries’ and ‘HPV16 genome integration does modulate intra-TAD host:host genome interactions and host gene expression’ to make the findings clearer to understand.*

Supplementary Figure 12A; the authors need to better describe in the figure legend what this panel is showing. It is not clear what is meant by “blue triangle” or what the comparison is between clone D2 and G2. Indicate the genomic position of the reference genome and the location of ARL15 and the integration locus. A colour scale should also be included.

*All the above changes have been made to the (now) Supplementary Figure 13 legend as requested and a more detailed explanation of the reduction in host:host interaction (differentially interacting region – DIR) between ARL15 and intergenic (MOCS2/FST) in clone G2 vs D2 is now present, as well as an overlaid ‘yellow triangle’ to highlight this change. Colour scales are also included for both Figs S13 and 15.*

Figure 8; The authors should provide further details of the distance from the integration site that genes are still differentially modulated in each subclone. For instance, what is the average size of the 5 gene bins and how does this relate to TADs/genomic architecture of the integration site?

*To improve the ability to compare variance data across all five clones analysed in one figure, previous Figure 8 and Figure S17 have now been merged into Figure 10. Details of the average size of the 5 gene bins (1.1Mb ±0.3Mb) (lines 425-427) and the distance that genes are still differentially modulated in clones G2 and D2 with extreme examples has now been added (lines 438-441), along with a more detailed description of the changes across individual clone integration sites (lines 431-438). Moreover, the interpretation of these data and relationship of variance to TADs/genomic architecture of the integration has now been further discussed in lines 566-583.*

Reviewer #3: (No Response)

*Thank you.*
